# Nanosized Bilosomes as a Potential Platform for Improved Therapeutic Efficacy of Capecitabine Against Colon Cancer in Rats: Formulation, Evaluation, and Optimization

**DOI:** 10.3390/ph19101531

**Published:** 2026-09-27

**Authors:** Mahmoud Elkot Mostafa, Abd El hakim Ramadan, Ahmed A. El-Shenawy, Islam Kamal, Loiy B. Hamed, Ahmed S. Saad, Ayman Salama, Mohamed Mahrous, Gamal M. K. Atwa

**Affiliations:** 1 Department of Pharmaceutics and Industrial Pharmacy, Faculty of Pharmacy, New Valley University, Al-Wadi Al-Gadid 72511, Egypt; mahmoudelkot.m@pha.nvu.edu.eg; 2 Department of Pharmaceutics, Faculty of Pharmacy, Port Said University, Port Said 42515, Egypt; abd.elhakim.ramadan@pharm.psu.edu.eg (A.E.h.R.); islamkamal@pharm.psu.edu.eg (I.K.); 3 Department of Pharmaceutics and Pharmaceutical Technology, Faculty of Pharmacy, Al Azhar University, Assiut Branch, Assiut 71524, Egypt; 4 Michael Sayegh Faculty of Pharmacy, Aqaba University of Technology, Aqaba 11191, Jordan; 5 Department of Pharmaceutics, Faculty of Pharmacy, East Port Said National University, Por Fouad 42526, Egypt; 6 Medical Emergency, Aqaba Modern Hospital, Aqaba 77110, Jordan; luaihamed99@gmail.com; 7 Department of Pharmacology and Toxicology, Faculty of Pharmacy, Port Said University, Port Said 42526, Egypt; a.saad@pharm.psu.edu.eg; 8 Pharmacology & Toxicology Department, Clinical Pharmacy Program, East Port Said National University, Port Said 42526, Egypt; 9 Department of Pharmaceutics, Faculty of Pharmacy, University of Tabuk, Tabuk 71491, Saudi Arabia; agrawan@ut.edu.sa; 10 Department of Biochemistry, Faculty of Pharmacy, Port Said University, Port Said 42515, Egypt; mohamed.mahrous@pharm.psu.edu.eg (M.M.); gamal.mohamed@pharm.psu.edu.eg (G.M.K.A.)

**Keywords:** nanosized vesicles, ultradeformable bilosomes, bile salts, capecitabine, colorectal cancer, histopathology

## Abstract

**Background/Objectives:** Colorectal cancer is a leading cause of global cancer-related mortality. Capecitabine, a 5-fluorouracil prodrug, effectively targets colorectal cancer but suffers from an extremely short half-life, requiring frequent administration. Bilosomes (membrane-stabilized bile salts) are vesicular systems that improve gastrointestinal stability, prevent drug leakage, and extend residence time. This study aimed to fabricate and characterize capecitabine-loaded nanosized bilosomes to overcome the pharmacokinetic limitations and systemic toxicities of conventional capecitabine therapy. By leveraging bile salt-stabilized vesicular systems, we seek to provide sustained capecitabine release, improve gastrointestinal permeability, and enhance therapeutic efficacy against colorectal cancer. We also assessed the anticancer potential of capecitabine-loaded nanosized bilosomes by measuring diagnostic and prognostic biomarkers, including cancer embryonic antigen, carbohydrate antigen 19.9, matrix metalloproteinase-9, and vascular endothelial growth factor A, to evaluate their impact on tumor progression, extracellular matrix degradation, and angiogenesis. **Methods:** In a 3^2^ full factorial design, nine capecitabine-loaded bilosomes were generated utilizing a central composite design within the framework of response surface methodology. Entrapment efficiency, in vitro drug release and its kinetics, vesicle size, zeta potential, and their kinetics were evaluated. The optimized capecitabine-loaded nanosized bilosomes formulation was subjected to further investigations, such as Fourier transform infra-red spectroscopy, differential scanning calorimetry, X-ray diffractometry study, stability studies, pharmacokinetic study, and in vivo studies, including detection of matrix metalloproteinase-9 and vascular endothelial growth factor A by Real-Time polymerase chain reaction, evaluation of colon biomarker, and examination of colon tissue through histopathology. **Results:** The prepared capecitabine-loaded bilosomes were nanosized spheres with suitable entrapment efficiency and a high zeta potential. After treatment with capecitabine-loaded bilosomes, the data collected from living organisms indicated a notable reduction in the serum values of carbohydrate antigen 19.9 and cancer embryonic antigen, gene expression values of matrix metalloproteinase-9 and vascular endothelial growth factor A. Histopathological study showed almost complete restoration of colonic features with a lesser extent of epithelial lining and crypt dysplasia. **Conclusions:** The present study findings demonstrate that capecitabine-loaded bilosomes possess high anti-tumor activity against colorectal cancer.

## 1. Introduction

Cancer develops when cells in one area of the body multiply rapidly and uncontrollably [1]. Malignant cells can invade and destroy adjacent healthy tissue. After breast and lung cancer, colorectal cancer (CRC) is the third most frequent type of cancer worldwide and the third leading cause of cancer-related death [2]. Worldwide, about 1 million new instances of CRC are identified in both men and women each year. Out-of-control development in the colon, rectum, or cecum is its hallmark. In CRC, a cell from an adenomatous polyp acquires the malignant characteristics of uncontrolled growth, invasion of nearby organs, and metastasis due to accumulating genetic alterations in the colon [3]. CRC continues to be a condition with high morbidity and mortality despite improvements in prognosis and treatment [4]. Early-stage CRC detection and treatment with appropriate medication play a critical role in reducing CRC spread. A prodrug of 5-fluorouracil (5-FU), capecitabine (CAP), inhibits thymidine synthesis and causes apoptosis. After consumption, the thymidine phosphorylase enzyme in tumor tissue converts CAP to 5-FU [5]. Compared to infusional FU, CAP is safer and has far fewer adverse effects, such as nausea, diarrhea, and alopecia. Myocardial infarction, hand-foot syndrome, angina, anemia, thrombocytopenia, and hyperbilirubinemia are among the negative effects of taking CAP in a traditional dosage form [5]. Rapid 5-FU clearance from the bloodstream also lowers CAP levels at the tumor site, thereby reducing therapeutic efficacy and, occasionally, causing severe side effects [6].

CAP is a class I drug with an extremely short half-life (38–45 min), so it must be administered more often. To overcome the above problems, various nanosystems have been developed, including niosomes, liposomes, virosomes, transferosomes, bilosomes, ethosomes, microspheres, nanoparticles (NPs), and polymeric micelles [7]. The benefits of nanosized vesicular systems include prolonged drug existence in the systemic circulation, decreased drug toxicity (the drug is delivered directly to the site of action), increased bioavailability (particularly for poorly water-soluble drugs), the ability to encapsulate hydrophilic and hydrophobic drugs, and protection of the entrapped moieties from metabolism with sustained drug action [8]. Bilosomes (membrane stabilized bile salts) are one of the most important vesicular systems that address the limitations of other NPs, such as degradation in the gastrointestinal tract (GIT) and limited permeability through the gastrointestinal membrane. This is due to the presence of bile salts like sodium deoxycholate (SDC) as a major component of the bilosomes. SDC helps enhance membrane permeability, protect active ingredients from natural bile salts, decrease leakage, and prolong their presence in the GIT by sticking to intestinal mucus. This process offers a solution to the issue of short half-life [9].

Among these biomarkers, crucial in diagnosis, prognosis, and treatments of CRC, are cancer embryonic antigen (CEA), carbohydrate antigen 19.9 (CA 19.9), matrix metalloproteinase-9 (MMP-9), and vascular endothelial growth factor A (VEGF-A). CEA is a glycoprotein generated during the embryonic stage, and its creation stops after birth. Hence, its concentration in serum is low and increases significantly during the development of cancer in adults, as cancer cells secrete CEA [10]. CEA is considered the common diagnostic marker for CRC in humans [11]. In addition, CA-19-9 has been identified as another important marker for CRC [12]. CA19.9 is a glycoprotein linked to the Lewis antigen found in red blood cells and produced by the pancreas in healthy individuals, colon, biliary duct, gastric, endometrial, and salivary epithelial cells [13]. The merging of CEA and CA 19.9 leads to a more precise biomarker for CRC [14]. MMPs are a family that can be ordered into several groups, including gelatinase, collagenase, matrilysin, and stromelysin. It is a zinc-dependent endopeptidases with more than twenty different members [15]. MMP-9 is extensively studied among MMPs and is a crucial enzyme that has a significant impact on numerous biological functions [16]. MMP-9 is involved in many biological processes due to its proteolytic activity in the extracellular environment, including the degradation of the extracellular matrix and the cleavage of cell-surface proteins [17]. MMP-9 is considered a potential biomarker for many cancers and plays an important role in diagnosis, monitoring, and disease treatment [18]. VEGF-A is an angiogenic growth factor that plays a vital role in mitogenesis and permeability. It regulates angiogenesis and vasculogenesis and plays a vital role in studies of tumor pathogenesis [19]. Essential for regulating vasculogenesis and angiogenesis, VEGF A stands out as the most widely investigated member of the VEGF family in studies exploring tumor progression [20].

Bilosomes, as nanosized flexible vesicular carriers composed of amphiphilic lipids, non-ionic surfactants, and bile salts (such as sodium deoxycholate), have emerged as a superior platform for oral cancer therapy. Unlike traditional liposomes and niosomes, the incorporation of bile salts into the lipid bilayer grants remarkable structural stability against enzymatic, acidic, and bile-induced degradation within the cruel GIT. In the context of CRC and GI oncology, nanosized bilosomes play a pivotal role in improving drug permeability across the intestinal mucosa, preventing premature drug leakage, and extending local and systemic residence times [21]. Recent studies have demonstrated the versatility of bilosomal nanocarriers in delivering diverse chemotherapeutic agents such as doxorubicin, methotrexate, and phytoconstituents, improving cellular uptake, enhancing cytotoxic activity against colon adenocarcinoma cell lines, and overcoming multidrug resistance mechanisms mediated by P-glycoprotein efflux pumps [21]. These structural and pharmacokinetic advantages make nanosized bilosomes a particularly promising vector for optimizing the oral bio-efficacy of capecitabine against CRC. The present study aims to fabricate and optimize capecitabine-loaded nanosized bilosomes (CAP-NBLs) to overcome the CAP pharmacokinetic limitations, rapid clearance, and adverse effects associated with conventional CAP therapy. We hypothesize that encapsulating CAP into bile salt-stabilized NBLs will improve drug intestinal permeability, prolong CAP presence in the GIT, and provide sustained release. This, in turn, is expected to potentiate the drug’s anticancer efficacy against CRC while mitigating systemic toxicity. To test this hypothesis, the therapeutic impact of CAP-NBLs will be investigated by monitoring crucial tumor biomarkers, specifically CEA, CA 19-9, MMP-9, and VEGF-A, which serve as key indicators of tumor burden, extracellular matrix degradation, and angiogenesis [22].

## 2. Results

### 2.1. Preparation of CAP-NBLs

Reversed-phase: Evaporation followed by ultrasound (ultrasonication) was used to prepare the CAP-NBLs. Primary practical trials were performed to investigate the effects of varying the amounts of Span 60 and SDC on the size of the vesicle and EE%. The trial showed that increasing the Span 60/cholesterol ratio to 5:1% *w*/*w* decreased vesicle size and increased EE%, consistent with previous studies [23]. The trial showed that an increase in SDC by more than 15% *w*/*w* markedly increased vesicle size and decreased EE%. Accordingly, we used Span 60/cholesterol ratios of 5:1, 4:1, and 3:1, and SDC at three levels below 15% *w*/*w*.

### 2.2. VS, PDI, and ZP

The mean VS of the CAP-NBLs ranged from 220.32 ± 1.39 to 412.45 ± 3.59 nm; the data are shown in Table 1. Increasing the concentration of Span 60 typically results in smaller bilosomal vesicles. Increased surfactant concentrations stabilize the vesicles and lessen vesicle aggregation. PDI values range from 0.139 to 0.422, suggesting uniform vesicle spreading, as shown in Table 1. The ZP values of all CAP-NBLs ranged between −32.6 ± 2.92 and −41.1 ± 1.18 mV. Figure 1 displays the ZP of the optimized CAP-NBLs (Table 1).

### 2.3. Drug Content

The drug content evaluation showed that the CAP amount in the formulated CAP-NBLs ranged from 85.56 ± 2.58 to 98.2 ± 3.02% (Table 1).

### 2.4. EE%

The EE% of the fabricated CAP-NBL formulations is shown in Figure 2. The EE% values of CAP-NBLs varied from 59.44 ± 1.12 to 87.66 ± 1.11%.

### 2.5. Yield Percentage

The obtained yield % results, shown in Figure 2, indicate that the yield % ranged from 85.56 to 98.20%.

### 2.6. In Vitro Release Studies

The in vitro release of CAP-NBLs in phosphate buffer at pH 6.8 is shown in Figure 3.

### 2.7. Mathematical Modeling of In Vitro Drug Release

All CAP-NBLs followed a Higuchi diffusion model, as indicated by the highest R^2^ value. Regarding the formulation of CAPB1, CAPB2, CAPB3, CAPB4, CAPB6, CAPB8, and CAPB9, the exponents (n) were less than 0.45, proving that the Fickian mechanism is the main mechanism governing the release of CAP from these formulations [24]. For the formulations coded with CAPB3, CAPB5, and CAPB7, the exponents (n) were greater than 0.45, proving that the non-Fickian (anomalous) mechanism is the main mechanism that controls the CAP release from these CAP-NBL formulations (Table 2).

### 2.8. Effect of the Independent Variables of CAP-NBLs on the Selected Responses

The response surface methodology was used to study the effect of Span 60 amount (X_1_:A) and SDC content (X_2_:B) on EE% of CAP (Y_1_), VS (Y_2_), and cumulative % CAP released at 24 h (Y_3_). The independent variables and the responses were modeled using second-order (quadratic) equations to demonstrate their relationship. The quadratic equation allows the evaluation of the combined and individual effects of the independent variables on the measured dependent variables (Y_1_, Y_2_, and Y_3_).

This model determines how each part, alone or in combination, affects results Y_1_, Y_2_, and Y_3_. When the coefficients are positive, the factors work well together; when they are negative, they tend to cancel each other out. We consider coefficients with a *p*-value less than 0.05 to be statistically significant.

The following equations were obtained from the factorial design, demonstrating the effects of the variables on Y_1_, Y_2_, and Y_3_ for CAP.

Y_1_ (EE, %) = +35.12 + 0.4503 Span 60 − 0.2000 SDC

Y_2_ (VS, nm) = +555.9138 − 2.6282 Span 60 + 1.6807 SDC

Y_3_ (% drug released at 24 h) = + 75.3600 + 0.26093 Span 60 + 0.1411 SDC

Span 60 (X_1_:A) had the most significant effect on increasing EE%, lowering VS, and increasing cumulative % drug released after 24 h, as demonstrated by the 3D response surface plots and the contour plots with higher positive coefficients, as it exceeded the critical significance value (*p* < 0.05) (Figure 4 and Figure 5). SDC (X_2_:A) had a significant effect on decreasing EE% and increasing VS and cumulative % released after 24 h, as shown by the 3D response surface plots and the contour plots with higher positive coefficients. The significant impact of Span 60 and SDC on the dependent responses is confirmed by the ANOVA results, where the Span 60 (A) and SDC (B) show highly significant F-value (116.24 for EE%, 65.46 for VS, and 60.5 for % released at 24 h), indicating a strong influence on the responses (Supplementary Materials, Tables S1–S3).

### 2.9. CAP-NBLs Optimization

The design software determines the optimal levels of the independent variables (Figure 6) based on the optimization results: 125 mg for A (Span 60 amount) and 10 mg for B (SDC amount), as listed in Table 3. Optimum CAP-NBLs were formulated and characterized for EE%, VS, and cumulative % drug released at 24 h.

The data obtained showed that the optimized CAP-NBLs had a mean VS of 243.35 ± 40.31 nm, a ZP value of −37.4 ± 2.18, and EE of 89.5 ± 2.33% and 43.8 ± 1.25% released after 24 h. Due to its close agreement with the predicted optimum and favorable CAP-NBLs formulation performance, this CAP-NBLs formulation was utilized for the subsequent characterization procedures.

### 2.10. Characterizations of the Optimized CAP-NBLs Formulation

#### 2.10.1. Stability Studies

The data on VS and EE% for CAP-NBLs over 42 weeks are illustrated in Figure 7. When the optimized CAP-NBLs formula was kept at 25 ± 2 °C for 43 weeks, a significant rise (*p* < 0.05) in VS from 240 ± 17.42 nm to 430 ± 15.2 nm was observed. Yet, when kept at 4 ± 0.5 °C, CAP-NBLs stayed constant, with no significant change in the VS after 42 weeks (247 ± 13.16 nm).

#### 2.10.2. TEM Micrograph of CAP-NBLs

TEM is an essential method for examining the appearance and surface properties of the optimized CAP-NBL formulation. The micrographs of the optimized CAP-NBLs exhibited spherical-shaped vesicles at the nanoscale, devoid of any vesicular fusion (Figure 8), which aligns with the findings from the dynamic light scattering (DLS) analysis (Figure 1A).

#### 2.10.3. DSC

Figure 9 shows the DSC thermograms of CAP, Cholesterol, SDC, egg yolk PC, Span 60, and the optimized CAP-NBLs formulation. DSC study revealed the presence of pure CAP in a crystalline state. The DSC analysis confirms the crystalline character of CAP, which exhibits a characteristic melting endotherm at 115.25 °C (Figure 9A) [25]. Cholesterol showed an endothermic peak at 149.3 °C (Figure 9B) [26]. SDC showed a small broad peak at 214.27 °C (Figure 9C) [27]. Egg yolk PC showed a characteristic endothermic peak at 51.57 °C (Figure 9D) [28]. Span 60 displayed two endothermic peaks at temperatures of 56.12 °C and 121.39 °C (Figure 9E) [29].

#### 2.10.4. ATR-FTIR

Figure 10 displays the ATR-FTIR spectra of the investigated samples. The ATR-FTIR spectra of pure CAP revealed a distinctive peak that included N-H stretching at 3523 cm^−1^, O-H stretching at 3242 cm^−1^, C-H stretching at 2926 cm^−1^, and C=O stretching at 1775 cm^−1^ [30]. Cholesterol’s ATR-FTIR spectra revealed prominent stretching vibration peaks for the –OH group at 3392.9 cm^−1^ and 2929.8 cm^−1^ for C–H stretching [31]. SDC’s ATR-FTIR spectra show both the alcoholic –OH group at 3338.3 cm^−1^ and asymmetric –CH3 stretching at 2929.5 cm^−1^ [32]. PC exhibits a broad peak at 3332.2 cm^−1^, indicating the existence of amine and alcoholic hydroxyl groups, along with the distinctive P-O group at 1735.4 cm^−1^ [33]. Conversely, Span 60 displays the ester carbonyl at 1735.4 cm^−1^ [34].

#### 2.10.5. XRD

The drug’s crystalline nature was confirmed by XRD analysis (Figure 11), which showed that CAP exhibited diffraction peaks at 2θ values of 19.28°, 20.68°, 26.08°, and 28.3°. Cholesterol peaks were observed at 13.9°, 16.8°, and 15.1° [35]. The peak of SDC is at 14.4° [36]. Additionally, Span 60 showed a peak at 21.3°, while PC showed peaks at 12.4°, 16.3°, and 19.9° [37].

#### 2.10.6. Ex Vivo Drug Permeation Assessment

The total CAP amount that crossed the membrane was 7200 ± 36.10 for CAP-NBLs and 2930 ± 57.50 µg/cm^2^ for pure CAP, as shown in Table 4 (*p* < 0.001). Consequently, compared to free CAP (29.3 ± 2.25%), the cumulative % of CAP that crossed the membrane from CAP-NBLs was 72 ± 1.42%, which is extremely significant (*p* < 0.001). Interestingly, compared to pure CAP, which has a permeation flux value (J_max_) of 2.44 ± 1.89 µg/cm^2^/h, the J_max_ of CAP-NBLs was 6 ± 1.78 µg/cm^2^/h. Furthermore, compared to pure CAP, the APC was measured to be 2.44 × 10^−4^ cm/min; the optimized CAP-NBL formulation had a much higher APC of 6 × 10^−4^ cm/min. Consequently, the ER of CAP-NBLs showed a 2.45-fold rise in drug permeability (Figure 12 and Table 4).

#### 2.10.7. In Vivo Pharmacokinetic Studies

Figure 13 shows the pharmacokinetic attributes of the CAP solution and the optimized CAP-NBL formulation following oral administration, and Table 5 shows the results of the investigated PKPs. At every treatment stage, the group that received CAP-NBLs had higher CAP plasma concentrations than the CAP solution group. Compared to CAP solution (4700 ± 105.12 ng/mL), CAP-NBLs showed a significantly higher (*p* < 0.001) C_max_ of 15,000 ± 272.48 ng/mL, indicating enhanced drug bioavailability [38]. The AUC_0–∞_ of CAP-NBLs was 165,230.99 ± 616.35 ng × h/mL, which was substantially more than the AUC_0–∞_ of CAP solution (27,703.86 ± 542.32 ng × h/mL) (*p* < 0.001). MRT ranged from 5.885 ± 0.54 to 9.009 ± 0.67 h, t_max_ ranged from 2 ± 0.1 to 6 ± 0.11 h, and the relative bioavailability of CAP-NBLs increased by 5.96-fold (*p* < 0.001) in comparison to the CAP solution.

#### 2.10.8. Gene Expression Levels of MMP-9 and VEGF-A by RT-PCR

Evidence clearly supporting the superiority of treatment with the optimized CAP-NBL formulation over the pure CAP solution was presented in the assessment of important colon cancer tumor markers, including MMP-9 and VEGF-A. As shown in the results, the gene expression level of colon MMP-9 and VEGF-A was markedly increased in the carcinogenic groups compared with the control groups (*p* < 0.001). Also, gene expression was significantly decreased in the group treated with the optimized CAP-NBL formulation compared with the carcinogenic groups and the group treated with CAP solution (*p* < 0.01 and *p* < 0.05, respectively), as illustrated in Figure 14A,B.

#### 2.10.9. Serum Levels of CA19.9 and CEA

In the current study, we observed that treatment with the optimized CAP-NBL formulation was superior to treatment with the corresponding pure CAP solution. This study measured specific colon cancer markers, CA19-9 and CEA, and found that serum levels of CA19-9 and CEA were significantly elevated in carcinogenic groups (*p* < 0.001) compared with control groups. Furthermore, treatment with the optimized CAP-NBL formulation showed significantly decreased serum levels of CA 19.9 and CEA compared with carcinogenic groups and the group treated with CAP solution (*p* < 0.01 and *p* < 0.05, respectively) (Figure 14C,D).

#### 2.10.10. Histopathological Examination

The evidence supporting the superior effect of the optimized CAP-NBL formulation over pure CAP solution is the histopathological examination of colon tissue. Photomicrograph A in Figure 15 shows a colon section from group 1 in a normal adult male Wistar rat, demonstrating normal histoarchitecture. Its mucosa was lined by columnar epithelial cells, and the crypts were lined by numerous goblet cells. Also, photomicrograph B in Figure 15 shows a colon section from group 2 in a normal adult male Wistar rat, taken with blank bilosomes, which shows the normal tissue structure, with both mucosa and crypts lined with columnar and goblet cells. Furthermore, photomicrograph C in Figure 15 shows a colon section from group 3, which shows marked epithelial lining and crypt dysplasia. Additionally, photomicrograph D in Figure 15 shows a colon section from group 4. The image shows hyperplasia of both mucosa and crypts. Photomicrograph E in Figure 15 shows a colon section from group 5, an adult male Wistar rat treated with CAP solution. The image shows decreased epithelial lining and crypt dysplasia, and some sections exhibit submucosal inflammatory cell infiltrates. Finally, photomicrograph F in Figure 15 shows a colon section from group 6, an adult male Wistar rat treated with CAP-NBLs, which shows almost complete restoration of colonic features with a lesser extent of epithelial lining and crypt dysplasia.

## 3. Discussion

For optimal stabilization and to avoid vesicle aggregation, a theoretical ZP value of ±30 mV is crucial [39]. Because bile salts have anionic properties due to the abundance of negatively charged groups in their structures, SDC significantly increases the negative ZP values [40]. The drug content analysis of the CAP-NBLs demonstrated efficient incorporation of CAP. This outcome highlights the suitability of Span 60, SDC, and the used excipient for co-delivery of CAP-NBL systems [41].

The obtained results of EE indicate a small amount of drug loss during formulation and effective drug encapsulation in the bilosomal nanosystem [42]. The following can be used to explain the phenomenon. First, the negative charge of the vesicle contributes to a significant repulsive force caused by the anionic nature of bile salts, which increases the size of the inner core of the bilosomes. Consequently, the bilosomes effectively trap hydrophilic drugs. Compared to other bile salts, the lower HLB value of SDC (16) makes it less hydrophilic and acts as a barrier, preventing drug leakage from the bilosomes. Furthermore, SDC can integrate into the bilayer membrane due to its surface-active properties, thereby increasing the membrane’s flexibility and the drug’s solubility. As a result, the EE rises [43]. Thirdly, compared with thin-film hydration methods, the drug encapsulation efficiency was significantly higher with the reversed-phase evaporation method [24]. Fourth, it was found that PC and cholesterol, when combined to form nanobilosomes, increased the % of EE of nanobilosomal vesicles, as cholesterol increases the stiffness and viscosity [44]. Examining the impact of surfactant concentration on EE, the results showed that surfactant concentration significantly affected EE% (*p* < 0.0001), with EE% increasing as surfactant concentration increased. Additionally, the amount of bile salt negatively affected EE%; increasing the amount of bile salt results in a decrease in EE% [30]. This problem arises when the vesicle’s lipid bilayer becomes overly fluid, allowing the drug to leak. Additionally, increased bile salt levels are linked to the formation of holes in the bilosomal membrane, which have been shown to possess a lower drug-loading efficiency than the initial vesicles [45]. The yield % values fell into the “very good” to “excellent” categories of experimental manufacturing, suggesting a very effective and successful procedure.

The in vitro release data showed that all CAP-NBLs exhibited a sustained release pattern compared to pure CAP. Also, as the concentration of Span 60 increased, the cumulative % of CAP release decreased from 59.6 ± 1.2% (CAPB1) to 47.33 ± 1.56% (CAPB5). The transition temperature of Span 60 is high. Increasing its concentration often results in a bilayer or surface that is less permeable and stiffer, thereby preventing the medication from diffusing [46]. Increased surfactant concentrations can make the formulation more viscous or cause more stable vesicles to form, thereby better retaining the drug and slowing its release into the release media and on the other hand, increasing the amount of SDC results in increased cumulative percent release, as illustrated by the increase from 49.32 ± 1.35% (CAPB6) to 53.31 ± 2.45% (CAPB9). This effect may be attributed to both SDC’s permeability-enhancing property and its impact on fluidity, which increases bilayer fluidity and permits drug release [47]. Secondly, the optimized CAP-NBLs were used for in vitro release studies in SGF for a duration of 2 h. Using pure CAP as a reference, the data are illustrated in the Supplementary Material, Figure S1. The cumulative % drug release was lower than that after 2 h in the case of SIF. This can be attributed to CAP’s weakly acidic properties, with a pKa of 8.8, which means it is highly soluble at higher pH values and less soluble at lower pH values [29]. However, all CAP-NBLs exhibit sustained release at pH 1.2, indicating that they are stable under GIT conditions [48].

The SDC negative charge is responsible for the stability of CAP-NBLs by preventing aggregation and fusion [49]. At 25 ± 2 °C, the EE% of CAP-NBLs also decreased from 89.5 ± 2.33% to 46.11 ± 3.3%. During storage of the optimized CAP-NBLs at 4 ± 0.5, EE% decreased insignificantly (Figure 7). At 25 ± 2 °C, CAP-NBLs possess enhanced Brownian motion (collision of bilosomal particles) gained from the heat energy of the surroundings, leading to the vesicles sticking together to form distorted bilosomal particles that are bigger in size and have a lower EE% [50]. Therefore, storing CAP-NBLs at cold temperatures is recommended to avoid merging and drug spillage during storage. The results obtained were consistent with those reported previously [51]. The absence of CAP crystals in TEM images indicates that CAP has completely changed to an amorphous rather than a crystalline state [52].

The DSC of the optimized CAP-NBL formulation showed a total absence of the characteristic peaks of CAP, which revealed that it was entirely enclosed within the fabricated vesicles (Figure 9F) [53].

Regarding ATR-FTIR spectra of the investigated samples, the optimized CAP-NBLs showed all of the distinctive CAP absorption peaks with little variation in amplitude and intensity, highlighting the insignificant physicochemical interaction between the medication and excipients [54]. All of the CAP’s prominent XRD peaks in the optimized CAP-NBL formulation faded, leaving only small, weak cholesterol peaks. This demonstrated that the drug was effectively loaded into the bilosomal vesicles and verified that it had completely changed from its crystalline to amorphous state, as shown in Figure 11 [54].

To determine how well CAP from CAP-NBLs can pass through the intestines compared to pure CAP, the ex vivo drug permeation study was conducted using a rat intestine for 120 min to assess gut permeation. The ability of bilosomal nanocarriers to improve CAP penetration through the GI membrane is shown by the permeation patterns shown in Figure 12. The smaller size and the efficient entrapment of CAP into the vesicles containing bile salts (SDC) are responsible for the increase in penetration [9]. According to previous studies, the negative charge of the bile salts imparts vesicles with elasticity and improves permeability across the intestinal membrane [44]. As a result, it makes it easier for the drug to diffuse across the membrane. Additionally, the vesicles’ negative charge enhances the absorption of the drug via the intestinal lymphatic transport system and promotes drug uptake by intestinal M-cells [44].

The optimized CAP-NBLs formulation had much higher C_max_ and AUC_0–∞_ caused by the effective encapsulation of CAP in the bilosomal vesicles, small VS, and avoiding hepatic metabolism [55]. The prolonged MRT and the delayed tmax may ensure sustained release from the investigated nanosized bilosomes. The enhanced relative bioavailability of CAP-NBLs in comparison to the CAP solution, which may have resulted from significant uptake of bilosomes by the M-cells in the GIT and their movement via the cell membrane with the aid of carriers [56]. Additionally, bilosomes’ exceptional stability in the GIT may enhance their bioavailability by protecting the delivery mechanism from degradation by bile salts [43].

The results of gene expression levels of MMP-9 and VEGF-A by RT-PCR agree with the study, which showed that MMP-9 plays a significant role in the progression of CRC due to its ability to degrade components of the extracellular matrix and basement membrane [57]. Additionally, in agreement with the study, which reported that MMP-9 degrades the extracellular matrix, it releases growth factors and reactivates factors. It plays an important role in progression, angiogenesis, rapid tumor growth, and metastasis [58]. These findings also corroborate a study that found VEGF-A is identified early in the progression of CRC and that its expression plays a vital role in metastases. It is linked to an excessive count of microvessels in colon cancer [59]. This is also in agreement with a study that showed that the VEGF family plays a vital role in the formation of blood vessels in embryonic development and in pathological angiogenesis and lymphangiogenesis, which permits tumor growth exponentially [60].

The serum levels of CA19.9 and CEA results agree with a study that reported that CEA protein concentration was significantly increased in DMH-induced CRC compared with the control group, as CEA is a cancer-specific marker [38,61]. This is also in line with a study that showed that CEA is a widely used tumor marker for early diagnosis of CRC, with a specificity of 78.8% for CRC [38]. This is additionally in agreement with a study that observed that low serum levels of CA-19-9 are found in normal cells and a significant increase in serum levels in neoplastic disease [38]. This is furthermore in agreement with a study that reported that the combination of both CEA and CA 19.9, when their serum levels are elevated, is an indicator of poor prognosis and tumor invasion and recurrence, which is important in the monitoring of CRC [12].

## 4. Materials and Methods

### 4.1. Material

SDC, CAP, and cholesterol were bought from Sigma-Aldrich Company (St. Louis, MO, USA). Egg yolk-derived phosphatidylcholine (PC) with a concentration of around 60%, confirmed through thin-layer chromatography (TLC), was obtained from Navi Mumbai, India, through Advent Chembio Pvt. Ltd. (Navi Mumbai, India). El-Nasr Co for Intermediate Chemicals Co., Assiut, Egypt, which provided sorbitan monostearate (Span 60), chloroform, diethyl ether, disodium hydrogen phosphate, and potassium dihydrogen phosphate. Visking dialysis tubing made of regenerated cellulose, with a diameter of 21 mm and a molecular weight cutoff from 12,000 to 14,000 Dalton, was purchased from Germany through Serva Electrophoresis GmbH Company located in Heidelberg. Each alternate chemical or reagent utilized was of analytical quality and employed without additional refinement.

### 4.2. Design and Preparation of CAP-NBLs

Nine CAP-NBL formulations were designed using a central composite design employing response surface methodology, and the outcomes of the experiments were assessed using Design-Expert^®^ software version 13.0.5.0 by Stat-Ease, Inc., located in Minneapolis, MN, USA [62]. Span 60 concentration (A) and sorbitol amount (B) were selected as the independent variables. % entrapment (EE%), vesicle size (VS), and CAP cumulative % released at 24 h were chosen as the dependent responses. Table 1 lists the suggested design. A slightly modified reverse-phase evaporation methodology was used to prepare CAP-NBLs. In a round-bottom flask (100 mL), a mixture of PC (100 mg), cholesterol (25 mg), and different levels of Span 60 was dissolved in a combination of chloroform and diethyl ether (1:1 *v*/*v*) using a hotplate/magnetic stirrer (Stuart UC152, Breckland Scientific Co., Hyde, UK), forming the non-aqueous phase. A water-based solution containing CAP (10 mg) and SDC in varying ratios was slowly added to the lipid mixture while being stirred on a magnetic stirrer at 3000 rpm, with a ratio of (1:3) of the organic mixture. Then, for twenty min, the mixture was sonicated until a milky *w/o* emulsion formed. The organic phase was vacuum-vaporized using a rotary evaporator for 30 min (Heidolph Instruments, Heidolph digital, GmbH & Co., Schwabach, Germany).

The final volume of the mixture was then adjusted by adding distilled water (the continuous phase) with stirring for an extra 10 min to complete the hydration. A homogenizer (high pressure) (IKA, T25, digital, Ho Chi Minh, Vietnam) was used to homogenize the crude dispersion for 10 min at 10,000 rpm to reduce particle size to the nanoscale. Next, ultrasonication was carried out with an ultrasonic probe sonicator (Sonics & Materials Inc., VCX 750, Newtown, CT, USA) for ten minutes in a chilled environment at an intensity level of 40%, alternating between being on for 59 s and off for 5 s. Until they were further characterized, the bilosomal dispersions were kept in a refrigerator at 4 °C [63]. Nine different formulations with different components were prepared (Table 6). The effects of Span 60 and bile salt concentrations on the physicochemical characteristics of the bilosomal formulations were evaluated.

### 4.3. CAP-NBLs Characterization

#### 4.3.1. Assessments of VS, Polydispersity Index (PDI), and Zeta Potential (ZP)

Using a Malvern Zetasizer (Ultra-red label, Malvern, Worcestershire, UK) at 25 ± 2 °C, VS, PDI, and ZP can be measured. The apparatus has a 90° scattering angle, a 120 s equilibration period, and a backscatter detection angle. To create a dispersion with a sufficient level of light scattering, 1 mL of CAP-NBLs dispersion was thinned with 10 mL of deionized water in a glass tube, then sonicated for ten min. Every measurement was done three times [64]. The mean ± standard deviation (SD) was employed to display the data.

#### 4.3.2. Estimation of Drug Content

An accurate volume (1 mL) of each formula (# 5 mg) of CAP was dissolved in ethyl alcohol, and the obtained solution was analyzed for the CAP amount spectrophotometrically at 301 nm using a double-beam spectrophotometer (Shimadzu 1601 Co., Kyoto, Japan) [65]. The drug content was determined from the following equation.

(1) $$\mathrm{CAP}\;\mathrm{content},\;\%=\frac{Actual\;CAP\;amount}{Theoretical\;CAP\;amount}\times 100$$

#### 4.3.3. EE%

Centrifugation/ultrafiltration was used to remove free CAP from CAP-NBLs to calculate the EE% [66]. Deionized water was used to dilute 1 mL of freshly prepared CAP-NBLs to 10 mL. To achieve effective separation, the diluted solution (3 mL) was transferred to a Micro Ultra centrifuge tube (3000 M.wt. cutoff) (Millipore, Burlington, MA, USA) and centrifuged at 8000 rpm for thirty min at 4 °C using a Centurion cooling centrifuge (K241R Pro, Scientific Laboratory Supplies, Nottingham, UK). The free CAP amount in the filtrate was measured spectrophotometrically at 301 nm by a UV-visible spectrophotometer, relative to deionized water [67]. The amount of free CAP was then subtracted from the initial amount, and the following equation was employed to assess EE%.

(2) $$\mathrm{EE}\;\%=\frac{Entrapped\;CAP\;amount}{Initial\;CAP\;amount}\times 100$$

#### 4.3.4. Yield Percentage

The subsequent equation was employed to calculate % of CAP yield after lyophilization of the prepared CAP-NBLs [68].

(3) $$\mathrm{Yield}\;\%=\frac{Weight\;of\;CAP-NBLs\;formulation}{Weight\;of\;CAP\;and\;ingredients}\times 100$$

#### 4.3.5. In Vitro Release Study of CAP

The cumulative % of in vitro release of CAP-NBLs and unloaded CAP, as a reference, was measured utilizing a shaking horizontal water bath and the dialysis bag technique in semi-gastric fluid (SGF) and semi-intestinal fluid (SIF) [63]. First, a dialysis bag made of a semipermeable cellophane membrane with a diameter of 21 mm (12,000–14,000 Dalton molecular weight cutoff) was filled with CAP-NBLs (equivalent to 5 mg CAP) and knotted at both ends with cotton threads. The dialysis bag was immersed in distilled water for the whole night before the procedures began. The systems were kept at a temperature of 37 ± 0.5 °C, and the dialysis pouch was placed in a 250 mL container filled with 100 mL of SIF (phosphate buffer pH 6.8, without enzymes). To verify the sink condition, a 5 mL sample of release medium was replaced with an equal amount of fresh phosphate buffer after 1, 2, 4, 6, 8, 10, 12, and 24 h from the start. Using phosphate buffer pH 6.8 as a blank, the amount of CAP released was determined spectrophotometrically at 301 nm. The average values were shown as in vitro total CAP % release over time (n = 3). Second, for further investigation, the in vitro release % of CAP-NBLs and CAP solution was evaluated in SGF (enzyme-free solution of HCl, pH of 1.2) for two h [63].

Various kinetic equations, such as zero-order (cumulative % left vs. time), first-order (log % substance left vs. time), Higuchi diffusion model (cumulative % substance discharged vs. square root of time), and Korsmeyer–Peppas model (log % substance discharged vs. log time), were used to analyze the in vitro release data of CAP-NBLs to comprehend the kinetics and mechanism of drug release. The straight line generated from analyzing the in vitro release data was utilized to determine the correlation coefficient (R^2^) for various kinetic models and the release exponent (n) values for the Korsmeyer–Peppas model [63].

### 4.4. The Optimized CAP-NBLs Characterization

#### 4.4.1. Stability of CAP-NBLs

For six months at 4 ± 0.5 °C and at room temperature, the optimized CAP-NBLs formulation was kept in vials (airproof, dry, clean, dark-colored borosilicate). The VS and EE% were examined and compared with the corresponding characteristics of the stored formulation at time zero. Each test was performed 3 times, and the mean ± SD was recorded [69].

#### 4.4.2. CAP-NBLs Morphology

The morphological characteristics and particle size of the optimized CAP-NBLs were evaluated by JEOL JEM-2100 Plus transmission electron microscopy (TEM), Akishima, Tokyo 196-8558, Japan, operating at 200 kV. Uranyl acetate was used to stain the chosen recipe. Before TEM scanning and visualization, the sample was dehydrated on the grid coated with carbon for 10 min at ambient temperature to produce a slender layer. Lastly, photos were captured at the proper magnification [52].

#### 4.4.3. Differential Scanning Calorimetry (DSC) Study

The Pyris 6 thermal analysis machine from PerkinElmer in Waltham, MA, USA, was employed to examine how CAP, SDC, cholesterol, Span 60, egg yolk PC, and the improved CAP-NBLs react to heat and to detect any alterations in the structure of CAP when enclosed in the bilosomes dispersion. The standard aluminum pans were then filled with the sample (2 mg) and heated from 30 °C to 300 °C at 10 °C/min [70].

#### 4.4.4. Attenuated Total Reflectance-Fourier Transform Infrared (ATR-FTIR) Spectroscopy

The possible chemical interactions between CAP and bilosome components can be detected by ATR-FTIR. Using an IR vortex spectrophotometer (Bruker, Ettlingen, Germany) connected to an ATR platinum diamond (an internal reflector diamond disc) with a 3500–500 cm^−1^ frequency range, 2.4 refractive index, and a resolution of 4 cm^−1^, the ATR-FTIR spectra of all samples were investigated [71].

#### 4.4.5. X-Ray Diffraction Pattern (XRD) Study

XRD was utilized to examine how encapsulation impacted the crystalline characteristics of the CAP powder. A diffractogram of CAP, SDC, cholesterol, Span 60, PC, and the optimized CAP-NBLs was recorded using an advanced X-ray diffractometer (Bruker instrument, D8, XRD, Yokohama, Japan) with the help of a radiation detector (monochromatized Cu Ka, Rigaku Corporation, Tokyo, Akishima, Japan) with a wavelength (λ = 1.54056 Å) operating at 40 kV and 40 mA to obtain two theta levels of 5° and 70° [72].

#### 4.4.6. Ex Vivo Permeation Assessment of CAP

Everted gut sac methodology was utilized, with minor modifications, to test both the CAP solution and the optimized CAP-NBLs to examine intestinal permeability of CAP from the optimized CAP-NBLs. After being fixed for the entire night and given diethyl ether anesthesia, the rats were sacrificed by cervical dislocation. After the small intestine was surgically removed, a 5 cm segment was cut and washed in normal saline to remove any remaining food particles. Using a smooth glass rod, the detached intestinal portion was turned over and given normal saline (2 mL) [73].

The extended gut pouch holding 10,000 μg of CAP, dispersed throughout the contained media, was placed in normal saline (50 mL) at 37 ± 0.5 °C, and the gut was supplied with 5% CO_2_ and 95% O_2_ [74]. Samples of 1 mL of the receptor compartment were removed at predetermined intervals (5, 10, 15, 30, 45, 60, 90, and 120 min) to determine the amount of CAP penetrated following appropriate dilution. A 0.45 μm pore size syringe filter was used to filter the withdrawn aliquots, and chromatographic analysis at 303 nm was used to assess the CAP concentration of either pure CAP solution or CAP-NBLs that had passed through the intestine [73].

Three trials were carried out. The cumulative % of CAP permeated from the CAP solution and the optimized CAP-NBL was estimated and plotted vs time (h). The apparent permeability coefficient (APC, cm/min) of the CAP solution and CAP-NBLs, and the enhancement ratio (ER) were calculated from the provided formulas [63].

(4) $$\mathrm{APC}=\frac{Q}{A\times C0\times t}$$

(5) $$\mathrm{ER}=\frac{APC\;of\;CAP-NBLs}{APC\;of\;CAP\;solution}$$

where Q: the CAP cumulative amount permeated (µg), C0: the initial CAP concentration (µg/mL), A: the surface area (cm^2^) of the intestinal sac, and t: the time period (min).

### 4.5. In Vivo Experiments

#### 4.5.1. Animals

The animal studies followed the ARRIVE guidelines and were authorized by the animal ethics committee at Zagazig University (ZU-IACUC), Approval number; ZU-IACUC/3/F/308/2026, at 25 March 2026. The experiments followed the regulations of the UK Animals (Scientific Procedures) Act, 1986, EU Directive 2010/63/EU, and the NIH Guide for the Care and Use of Laboratory Animals (NIH Publications No. 8023, revised 1978). Male Wistar rats (average weight = 150 g) and healthy New Zealand rabbits (average weight = 2.25 kg) were obtained from the faculty of veterinary medicine, Suez Canal University, Ismailia, Egypt. All animals were kept in a controlled environment (temperature, 20–22 °C; humidity, 50 ± 5%; night/day cycle, 12 h) with standard diet pellets and sterile water.

#### 4.5.2. Pharmacokinetic Studies

The animals were split into three groups (n = 6). Groups B and C were the tested groups, while group A served as the control and received only normal saline. Group B received a 10 mg/kg dosage of pure CAP solution by oral gavage, while group C received CAP-NBLs at the same dose level. After properly handling the rabbits, blood samples (0.5 mL) were drawn after 0.5, 1, 2, 3, 4, 6, 8, 10, 12, 18, and 24 h from the ear vein. The samples were then stored in heparin-coated microcentrifuge tubes and centrifuged (Primo Heraeus, Biofuge, Osterode am Harz, Germany) at 4000 rpm for 15 min to separate plasma. The resulting plasma samples were stored at −20 °C for further CAP analysis. A high-performance liquid chromatography (HPLC) system from Jasco Co., Hachioji-shi, Tokyo, Japan, featuring a dual pump and a UV detector model SPD-10A, was utilized for the chromatographic examination. A C-18 column (250 mm × 4.6 mm) served as the stationary phase, and a 50:50 *v*/*v* mixture of distilled water and acetonitrile (HPLC grade) served as the mobile phase. The run time was 15 min, and the lambda max was set at 303 nm. For 20 µL of the injected sample, the pump’s flow rate was 1 mL/min [75]. The Microsoft Excel program (Microsoft Office LTSC professional Plus 2024) was then used to examine several pharmacokinetic parameters (PKPs). The results were presented using the mean ± standard deviation for various parameters such as the area under the curve (AUC), maximum plasma level (C_max_), time taken to reach maximum plasma level (t_max_), elimination rate constant (K_el_), and mean residence time (MRT).

#### 4.5.3. Animal Experiments

Ninety male Wistar rats in good health were distributed randomly into six groups, with each group containing 15 rats after a 2-week adaptation period. Animals in groups 1 and 2 were not exposed to any carcinogenic materials. They received 0.5%, *w*/*v* carboxy methylcellulose sodium (CMC-Na) in water and blank bilosomes every day by oral route during the experimental period and were considered as the negative control groups. A single injection of dimethylhydrazine (DMH) (20 mg/kg, subcutaneously) for four weeks, twice weekly, was administered to animals in groups 3, 4, 5, and 6 to enhance the cancer-causing impact of DMH [76]. The 4-week protocol of DMH administration was specifically selected to induce early-stage colorectal carcinogenesis, such as aberrant crypt foci (ACF) and early pre-neoplastic lesions, rather than fully developed macroscopic tumors. This timeline aligns with established models for studying early intervention or preventive effects [10,11,12,30]. In accordance with the dose was selected according to the previous study of Hassan, El-Kalaawy et al. 2023) [77]. Another study uses 180 mg/kg as Kobuchi, Arimoto et al. 2024 [78]. Animals in groups 3 and 4 were administered a mixture of CMC-Na in water (0.5%, *w*/*v*) and empty vesicles individually via oral gavage each day, starting two weeks post the final DMH dosage throughout the experiment, and were considered as positive control groups. Animals in group 5 were given CAP (200 mg/kg body weight) dissolved in a solution of CMC-Na in water (0.5%, *w*/*v*) daily by oral gavage for 8 weeks [79]. Animals in group 6 were given an equal amount of CAP from CAP-NBLs for 8 weeks, as shown in group 5. Another study reported that the using of different doses of CAP in for showing efficacy and cardiotoxicity observation of different doses of CAP in patient-derived tumor xenograft of mice with colorectal cancer using 200, 300, and 400 mg/kg CAP as low, middle and high doses [74]. Additionally, the median toxic dose (TD50) for capecitabine in rats can reach up to 359 mg/kg [80].

##### Serum Specimen Collection

Blood samples were obtained from the lateral tail vein of animals using needle cannulation after 12 h fasting interval following the latter dosage, and for coagulation of blood samples, wait for 20 min and then centrifuge at 4000 rpm at 4 °C for 15 min using a Beckman Show cooling centrifuge (Waltham, L3-50, MA, USA). The serum was collected and frozen at −80 °C [76].

##### Tissue Specimen

After cervical dislocation of rats under anesthesia using compressed CO_2_ gas in a cylinder with the inflow of gas to the induction chamber shown, the colon was thoroughly washed with phosphate-buffered saline solution, then wiped with tissue paper cut in half. The first part was frozen at −80 °C for later analysis by real-time polymerase chain reaction. The second part was kept in 10% formalin for histopathological examination [81].

##### Detection of MMP-9 and VEGF-A by Real-Time Polymerase Chain Reaction (RT-PCR)

The primers were acquired from Vivantis Technologies in Subang Jaya, Malaysia, and the reference sequences from NCBI were utilized to create the primers.

MMP-9 forward Tm = 60 °C: 5′-TCGGATGGTTATCGCTGGTG-3′.
MMP-9 reverse Tm = 60 °C: 5′-GCCACGACCATACAGATGCT-3′.
VEGF-A forward Tm = 60 °C: 5′-CCAGGACCTGAATTCCCAGC-3′.
VEGF-A reverse Tm = 60 °C: 5′-ACTCCCTAATCTTCCGGGCT-3′.
β-actin forward Tm = 60 °C: 5′-CCCGCGAGTACAACCTTCTT-3′.
β-actin reverse Tm = 60 °C: 5′-AACACAGCCTGGATGGCTAC-3′.

The steps of RT-PCR were as described by Atwa, Omran et al. [76]. The data were used to calculate fold variation and relative expression utilizing the 2^−ΔΔCT^ method [76]. β-actin was employed as the internal control gene.

##### Assessment of Colon Biomarkers

Serum levels of both CA 19.9 and CEA were assessed using an enzyme-linked immunosorbent assay kit provided by Glory Science (Hangzhou, China) [82].

##### Histopathological Evaluation

The tissues of the colon were preserved in formalin (10%, *v*/*v*) solution and subjected to a series of washes with ethanol for dehydration. Afterward, the specimens underwent xylene treatment, were immersed in paraffin at 56 °C using a hot-air oven for a full day, and then sliced into 4–5 µm segments. The sections were dyed using hematoxylin and eosin (H&E) and scrutinized under a light microscope (Olympus, Center Valley, PA, USA) by a histopathologist who was unaware of the intervention [83].

### 4.6. Statistical Analysis

One-way analysis of variance (ANOVA) was used to statistically analyze the following data: EE%, VS, ZP, drug content, and cumulative % CAP amount released after 24 h; data are presented as means ± SD. Statistical data analysis was conducted using GraphPad Prism version 5.0 (GraphPad, San Diego, CA, USA). Tukey’s *t*-test and analysis of variance were used to compare groups. A *p*-value of less than 0.05 was chosen as the significance level, and all pertinent findings were presented graphically as means ± SD.

## 5. Strengths and Limitations of the Study

While the obtained results demonstrate the clear clinical potential of CAP-NBLs, a balanced consideration of the present investigation’s strengths and inherent limitations provides necessary context for future translation.

A primary strength of the present study lies in the NBLs formulation design and thorough multi-level evaluation. By employing a 3^2^ full factorial design with response surface methodology, we successfully optimized a stable nano-bilosomal vesicles platform that overcome the biopharmaceutical limitations of CAP. Significantly, CAP-NBLs achieved a 2.45-fold increase in ex vivo membrane permeability and a 5.96-fold enhancement in oral relative bioavailability compared to free CAP solution. The obtained in vivo pharmacokinetic results were directly reflected in the in vivo setting, where CAP-NBLs treatment yielded marked downregulation of the assessed oncogenic and angiogenic markers (MMP-9 and VEGF-A), suppressed serum biomarkers (CEA and CA 19-9), and demonstrated near-complete recovery of colonic architecture upon histopathology.

Contrariwise, specific limitations deserve attention. First, the rat model successfully demonstrated anti-tumor efficacy and systemic absorption; however, pronounced interspecies variations in GI fluid dynamics, transit durations, and metabolic enzyme profiles prevent the direct translation of these pharmacokinetic results to humans without prior clinical assessment. Second, a temperature-dependent stability study revealed that ambient storage of the optimized CAP-NBL formulation showed significant vesicular enlargement over the study time, confirming that a cold-storage condition is mandatory to preserve BLs integrity. Lastly, although various molecular and histopathological markers were investigated, comprehensive toxicity profiling including chronic hepatic and renal function panels and longitudinal survival rate analyses remained beyond the immediate scope of this investigation. Accounting for these points within orthotopic or patient-derived xenograft models is urgent for the comprehensive validation of the fabricated CAP-NBLs before the clinical trials.

Although comprehensive serum pharmacokinetics, colonic biomarker suppression, and histopathology confirmed systemic absorption and local therapeutic efficacy, organ-specific tissue distribution studies were not conducted in this preliminary phase. Because CAP is a prodrug requiring multi-step enzymatic conversion to 5-FU, quantifying CAP versus active metabolite accumulation in target colonic tissue and off-target organs (e.g., liver, kidneys, and heart) remains an essential next investigation step. Incorporating quantitative tissue distribution and organ-toxicity profiling in future studies will be pivotal to fully elucidate the biodistribution pattern and safety margins of CAP-NBLs prior to clinical trials.

## 6. Conclusions

In the present study, CAP-NBLs were prepared to overcome the drawbacks associated with infusional 5-FU, traditional CAP formulations, and CAP’s extremely short half-life. The prepared CAP-NBL formulations were nanoscale spheres with suitable entrapment efficiency, higher ZP values, and sustained release behavior. ATR-FTIR analysis showed no interaction between CAP and the excipient. DSC data showed no CAP-specific peak, indicating that CAP was successfully encapsulated into the bilosomal matrix without significant interaction or degradation. The XRD confirms the transition of CAP from crystalline to amorphous form upon encapsulation into bilosomes. The in vivo data after treatment with CAP-NBLs showed that the CA 19.9 and CEA serum levels were markedly decreased, gene expression levels of MMP-9 and VEGF-A, and a histopathological study showed almost complete restoration of colonic features with a lesser extent of epithelial lining and crypt dysplasia.

## Figures and Tables

**Figure 1:**
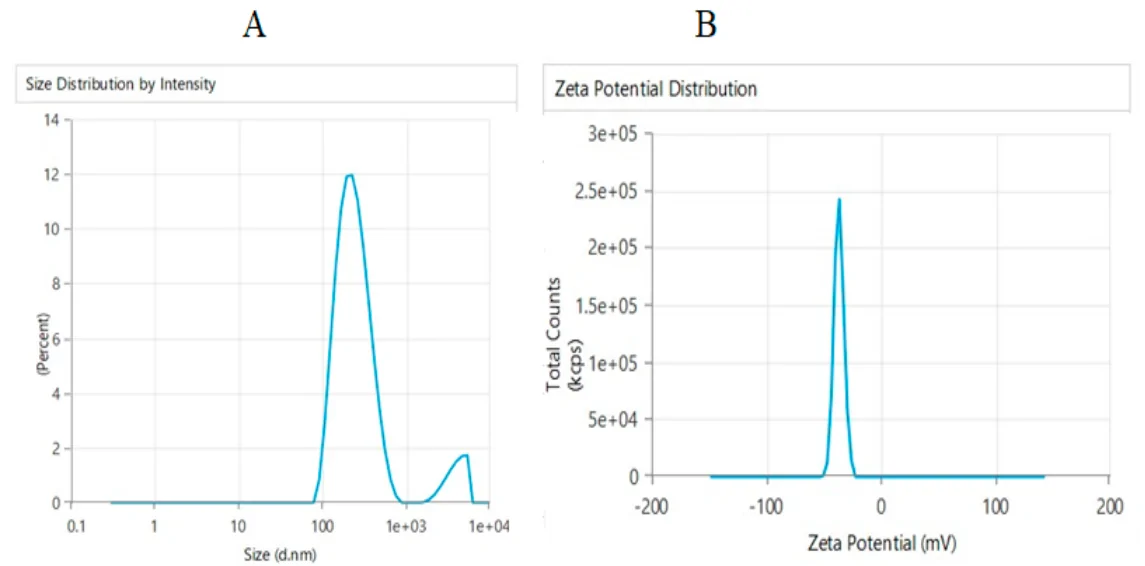
Particle size of the optimized CAP-NBLs (**A**) and its ZP (**B**).

**Figure 2:**
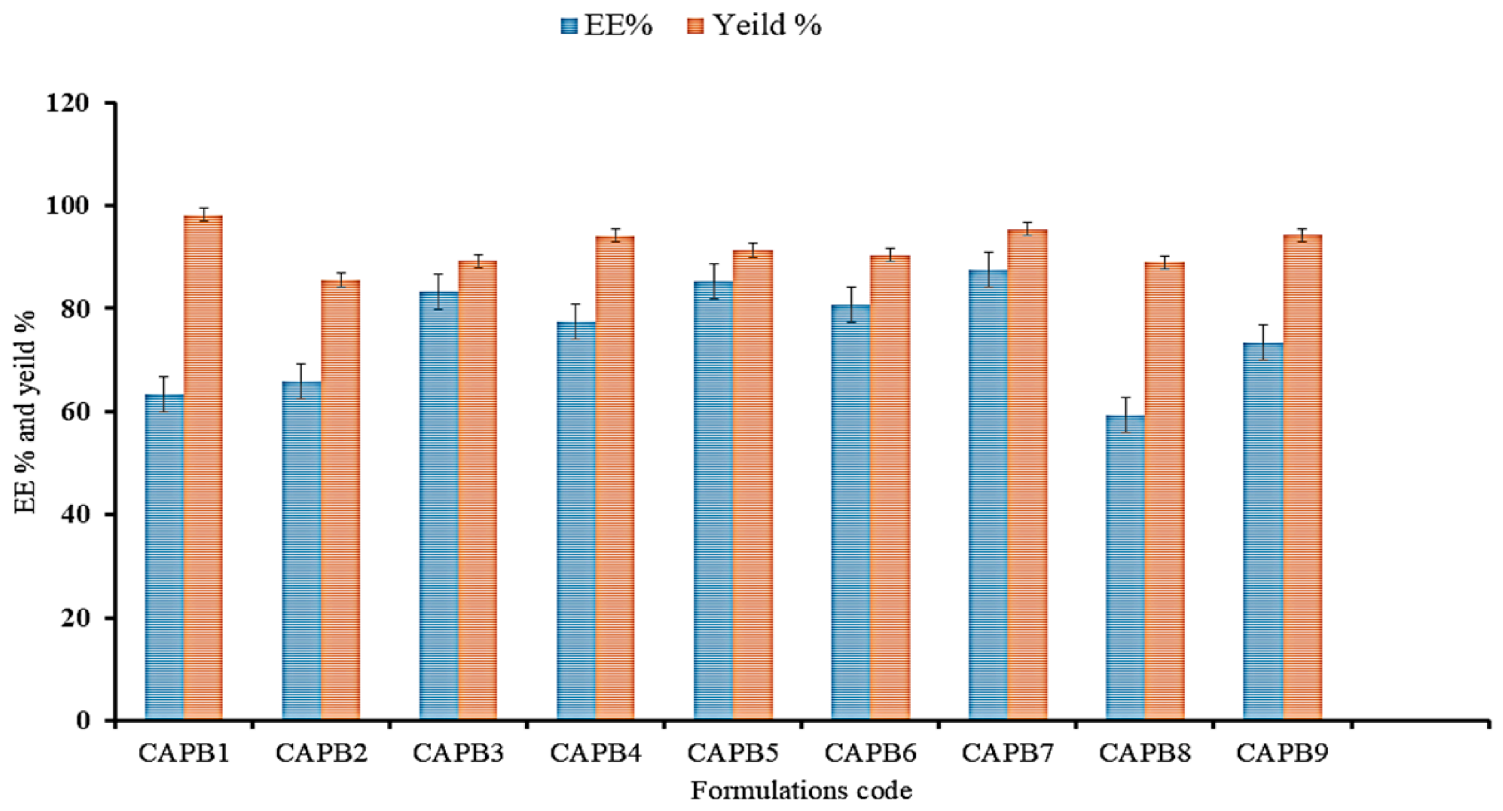
EE% and yield % of the various CAP-NBL.

**Figure 3:**
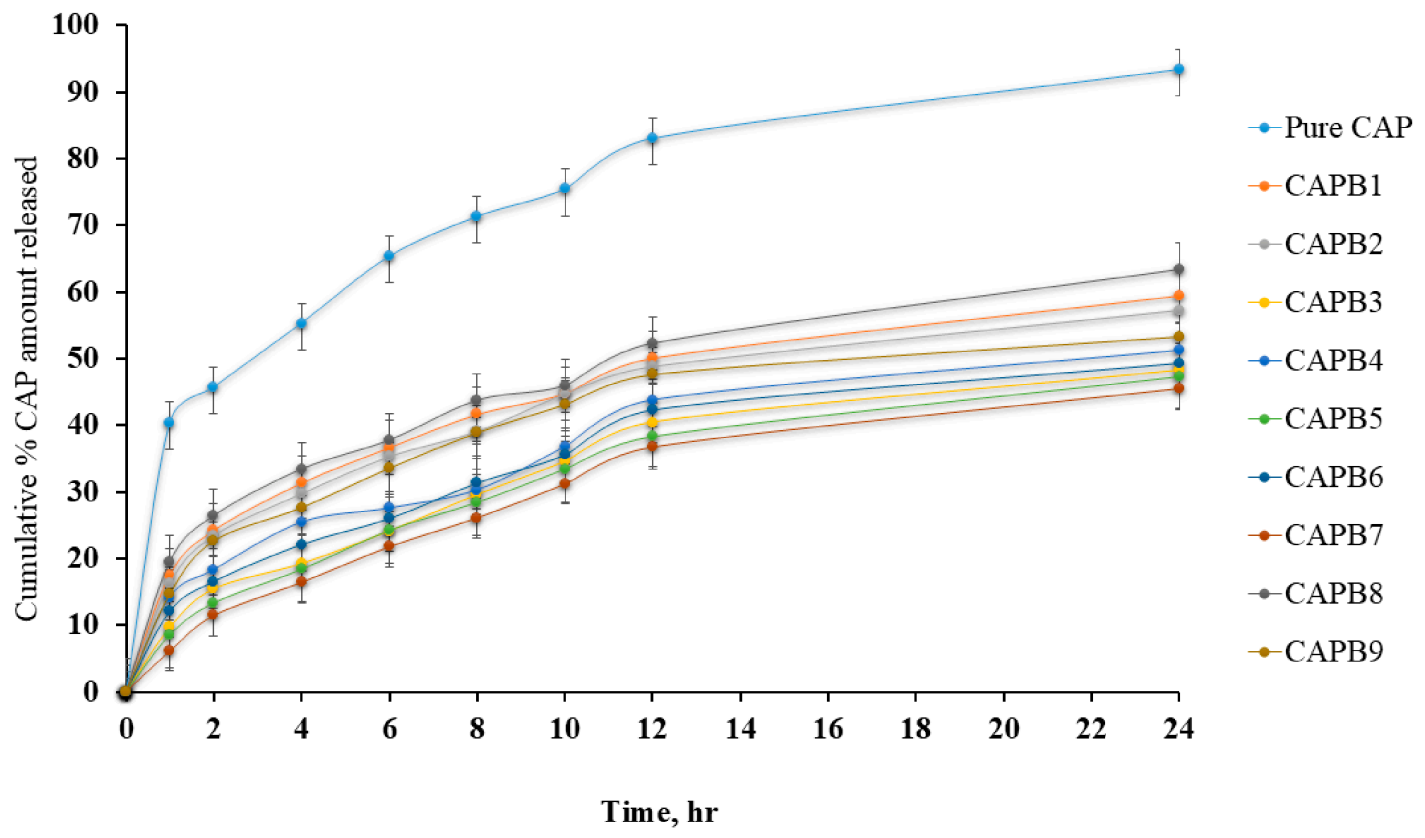
CAP in vitro release pattern in phosphate buffer pH 6.8, compared to the pure CAP in the same media.

**Figure 4:**
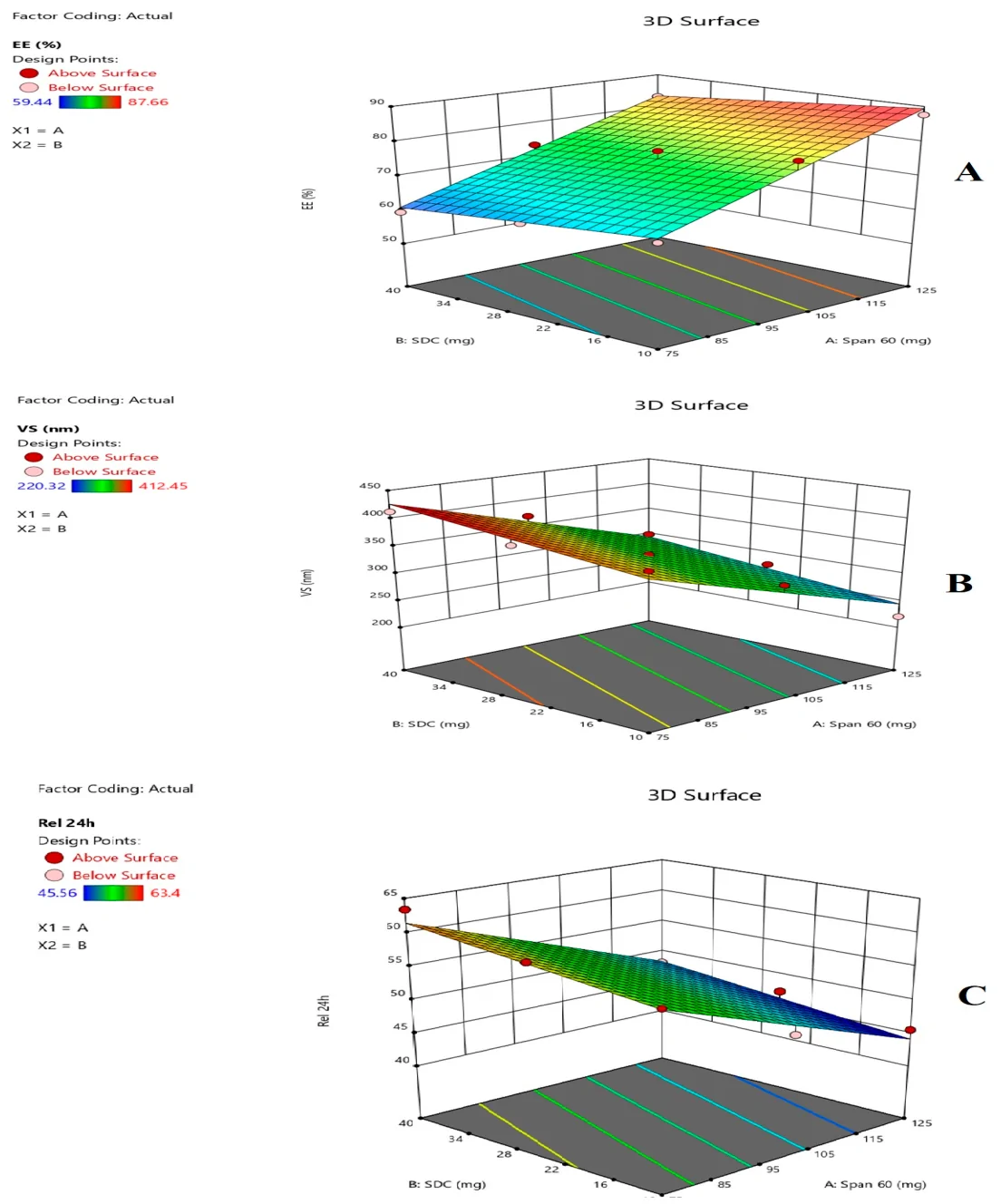
3D surface plots show how independent variables impact EE% (**A**) and VS (**B**), and cumulative % drug released at 24 h (**C**).

**Figure 5:**
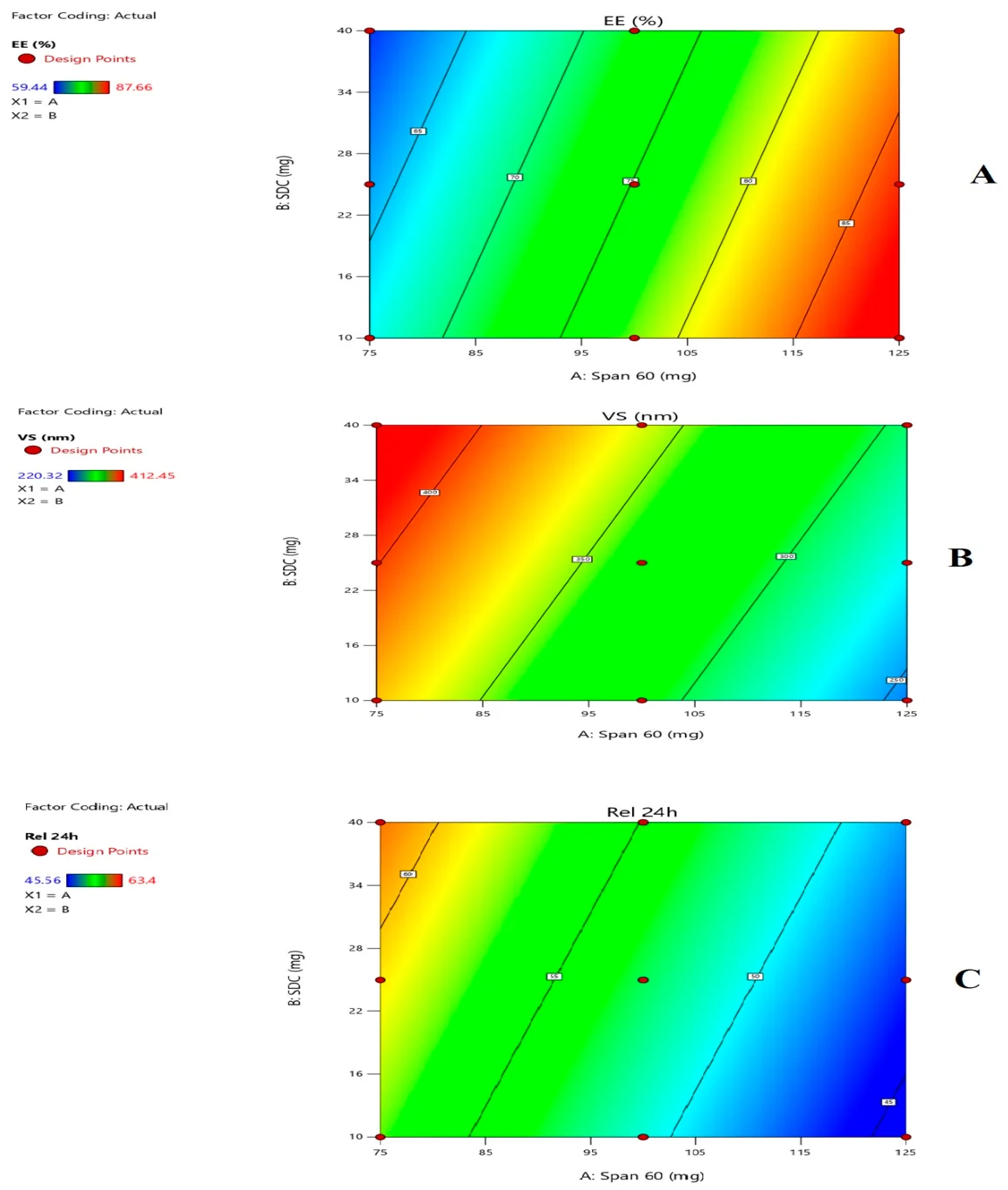
Contour graphs predicting how independent variables impact the EE% (**A**), VS (**B**), and cumulative % drug released after 24 h (**C**).

**Figure 6:**
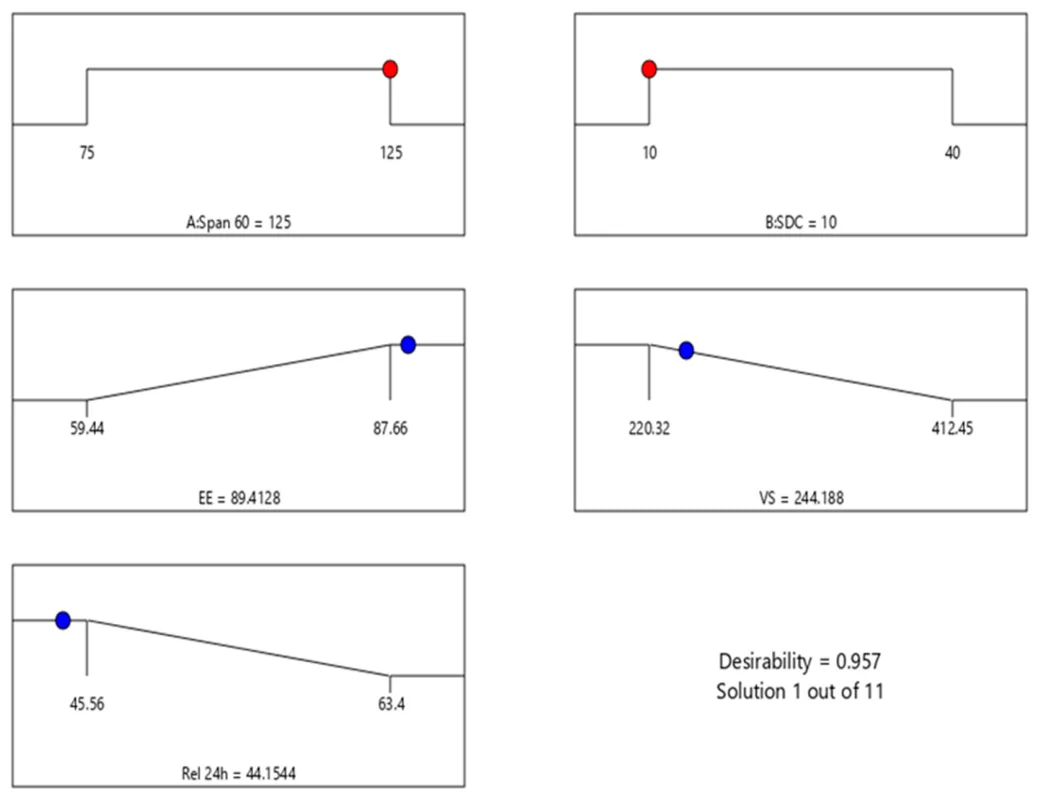
The structure and the variables that rely on the optimized CAP-NBLs.

**Figure 7:**
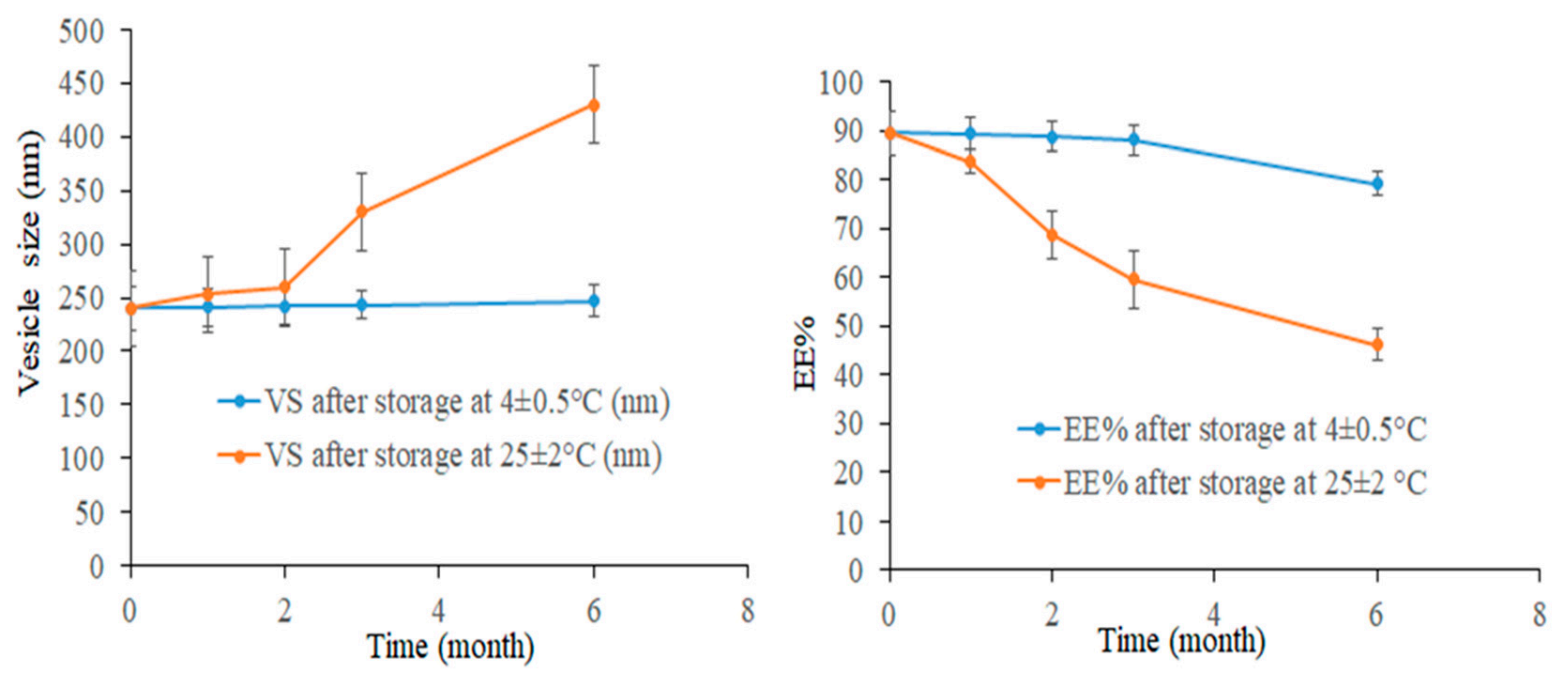
Stability profiles of the optimized CAP-NBLs formulation stored at 4 °C and 25 °C for 42 months (mean ± SD, n = 3).

**Figure 8:**
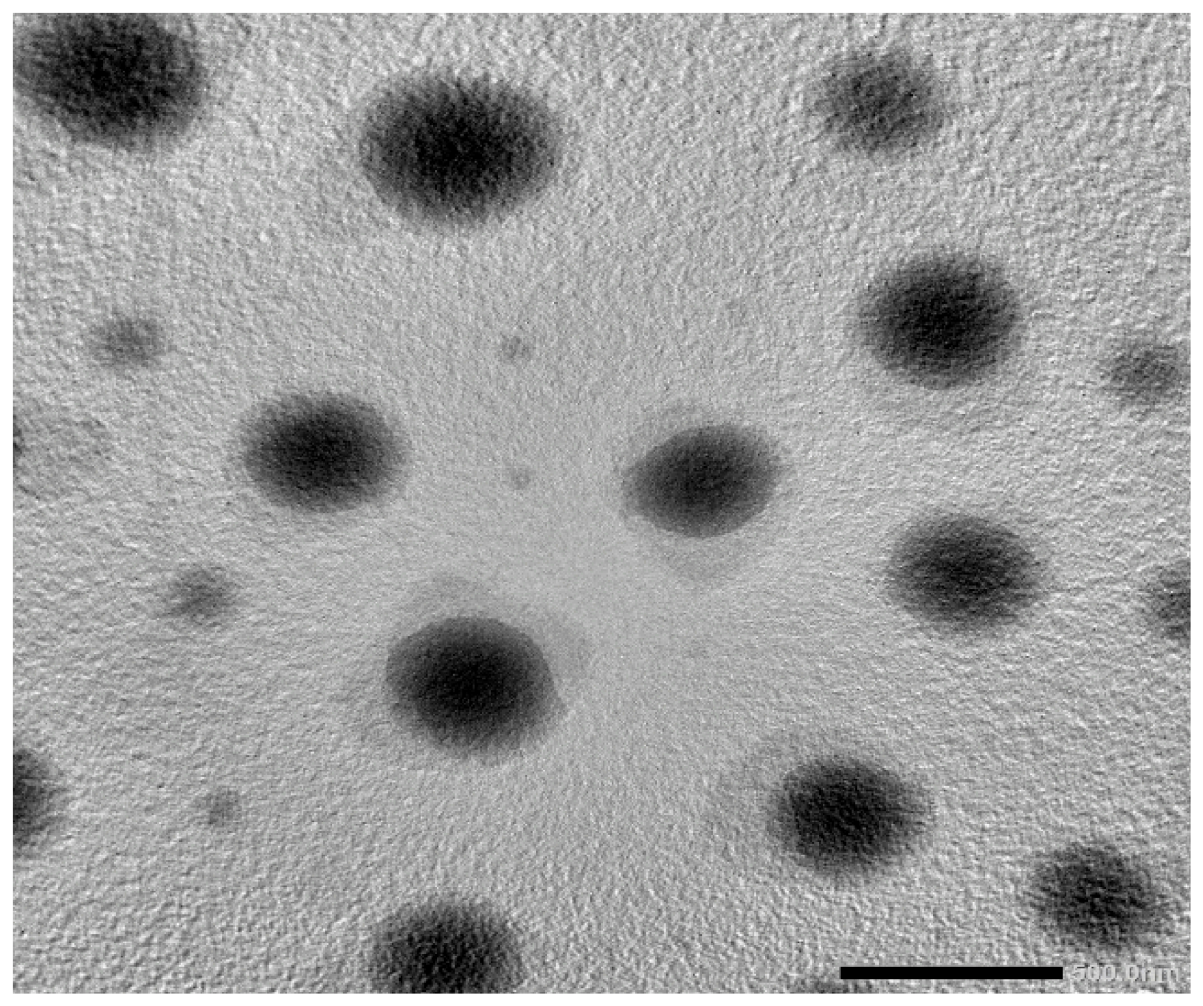
TEM micrograph of the optimized CAP-NBLs.

**Figure 9:**
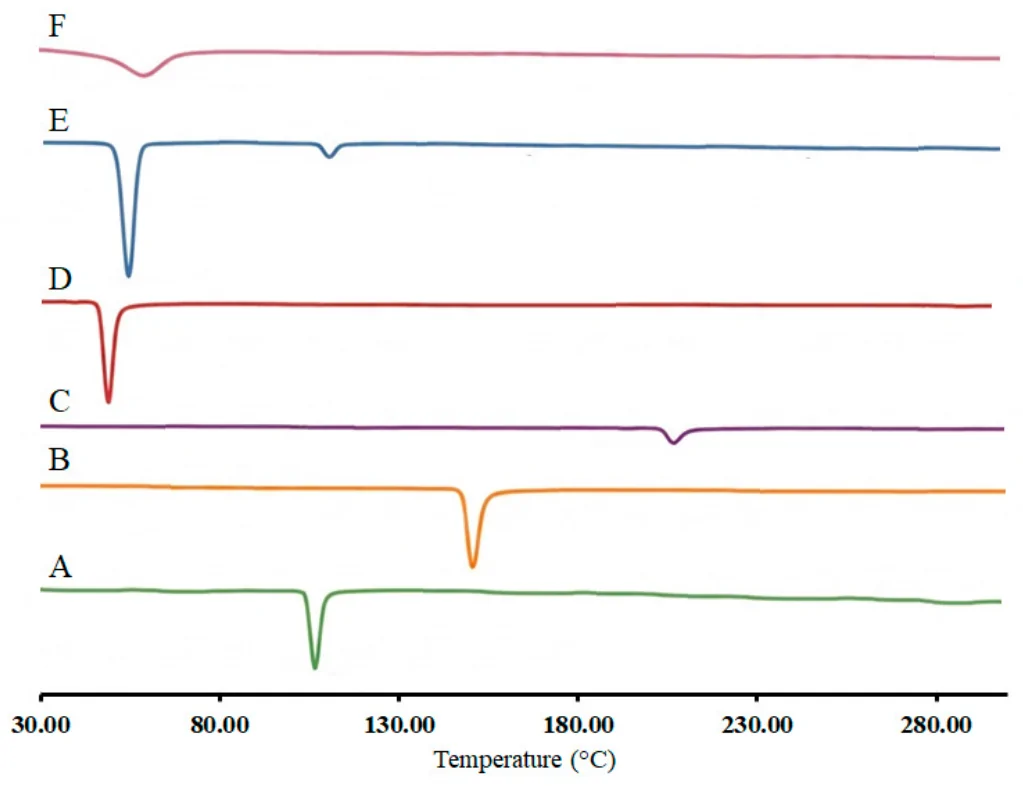
Thermal analysis graph showing (**A**) CAP, (**B**) cholesterol, (**C**) SDC, (**D**) PC, (**E**) Span 60, and (**F**) the optimized CAP-NBL formulation.

**Figure 10:**
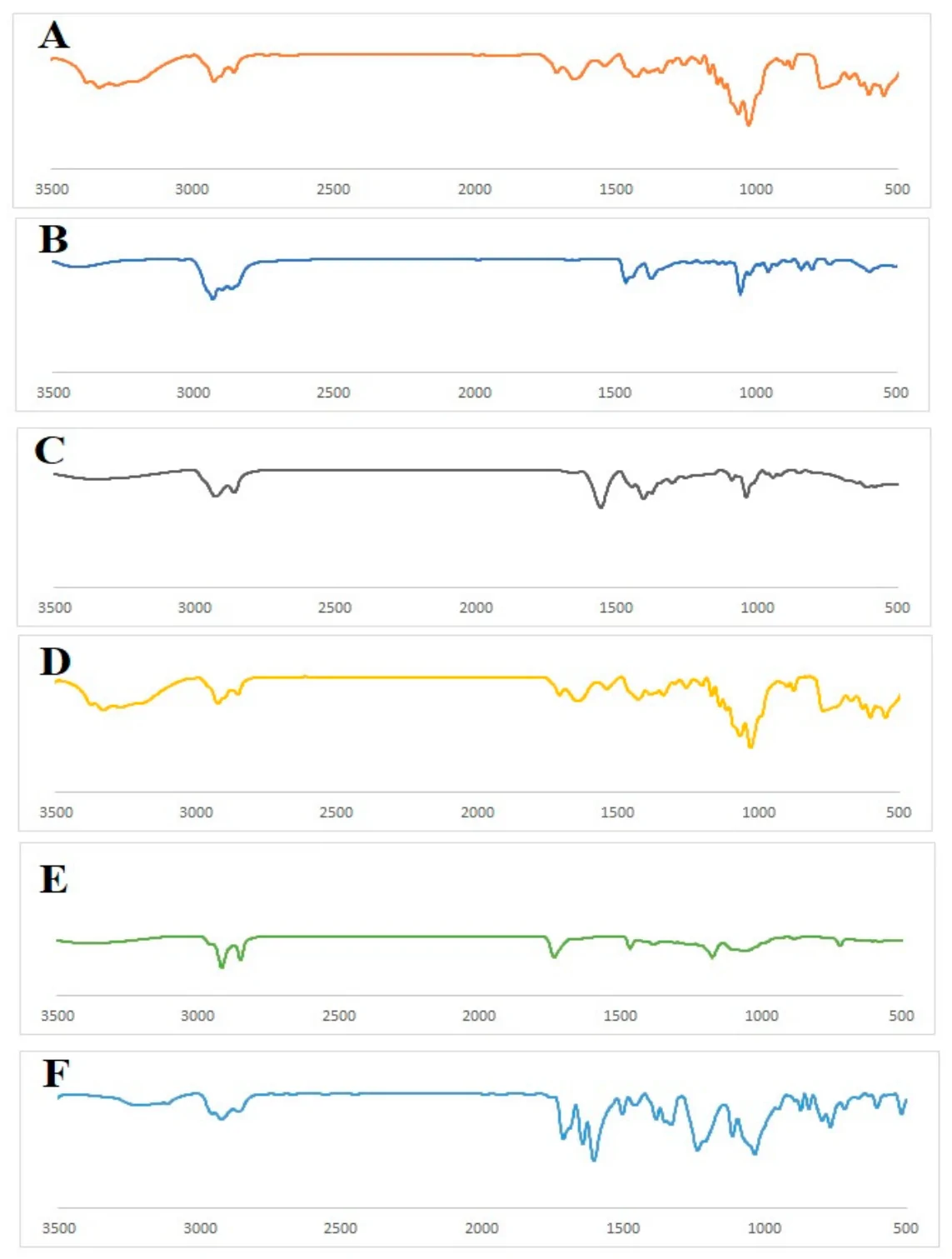
ATR-FTIR spectra of (**A**) CAP, (**B**) cholesterol, (**C**) SDC, (**D**) PC, (**E**) Span 60, and (**F**) the optimized CAP-NBL formulation.

**Figure 11:**
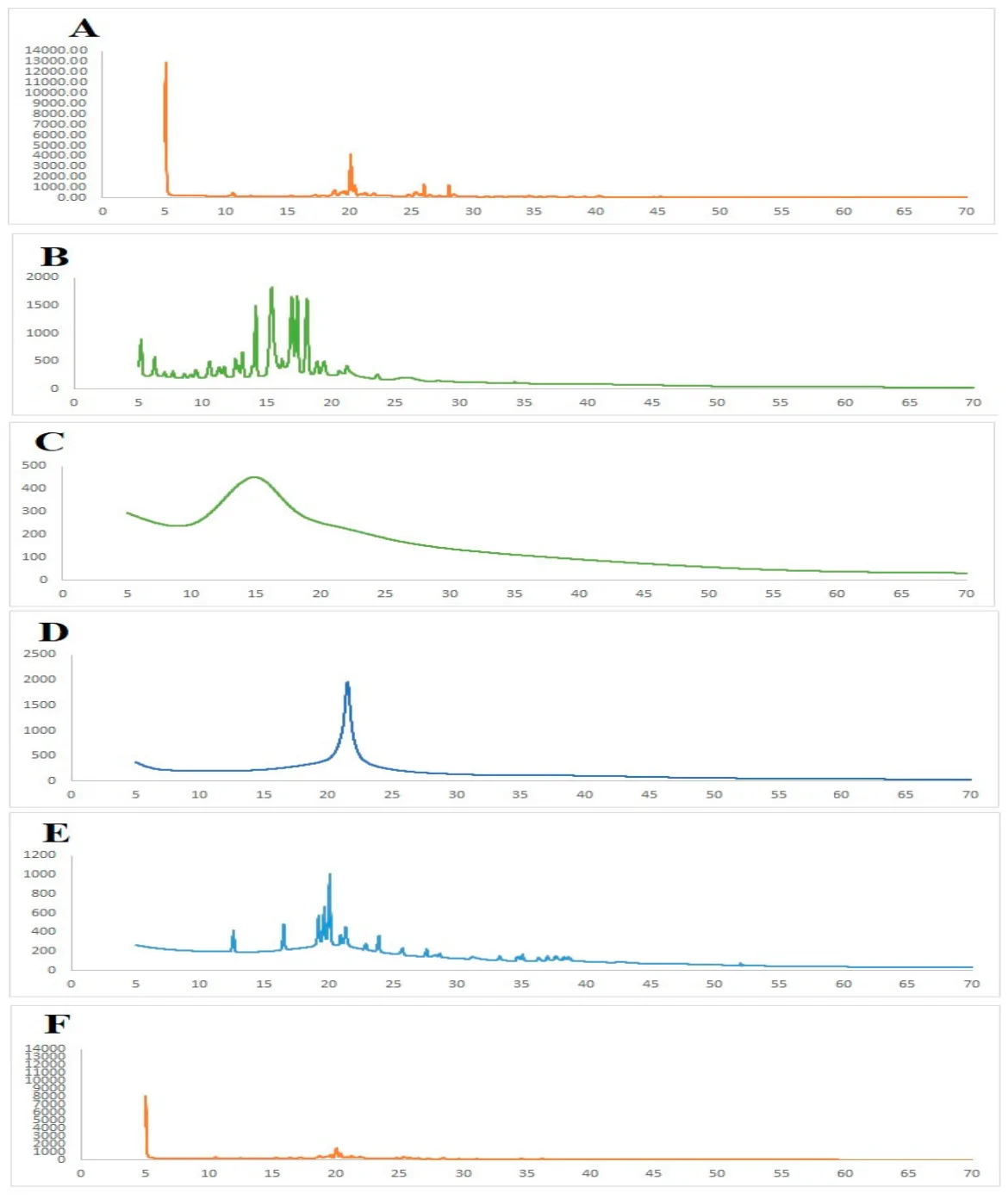
XRD analysis of (**A**) CAP, (**B**) cholesterol, (**C**) SDC, (**D**) Span 60, (**E**) PC, and (**F**) the optimized CAP-NBL formulation.

**Figure 12:**
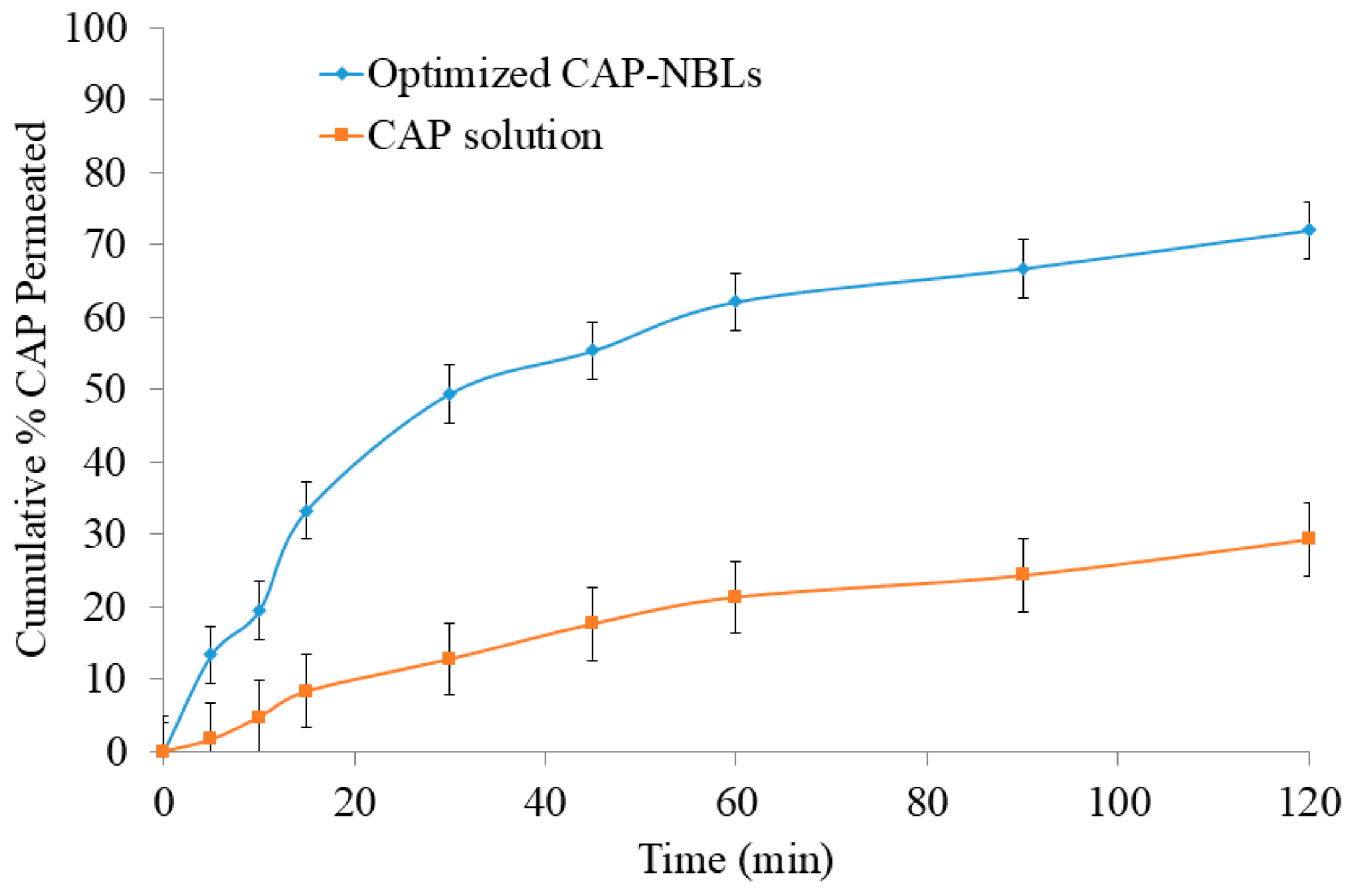
Ex vivo gut permeation investigation showing cumulative % of CAP permeated against time from the CAP solution and the optimized CAP-NBL formulation.

**Figure 13:**
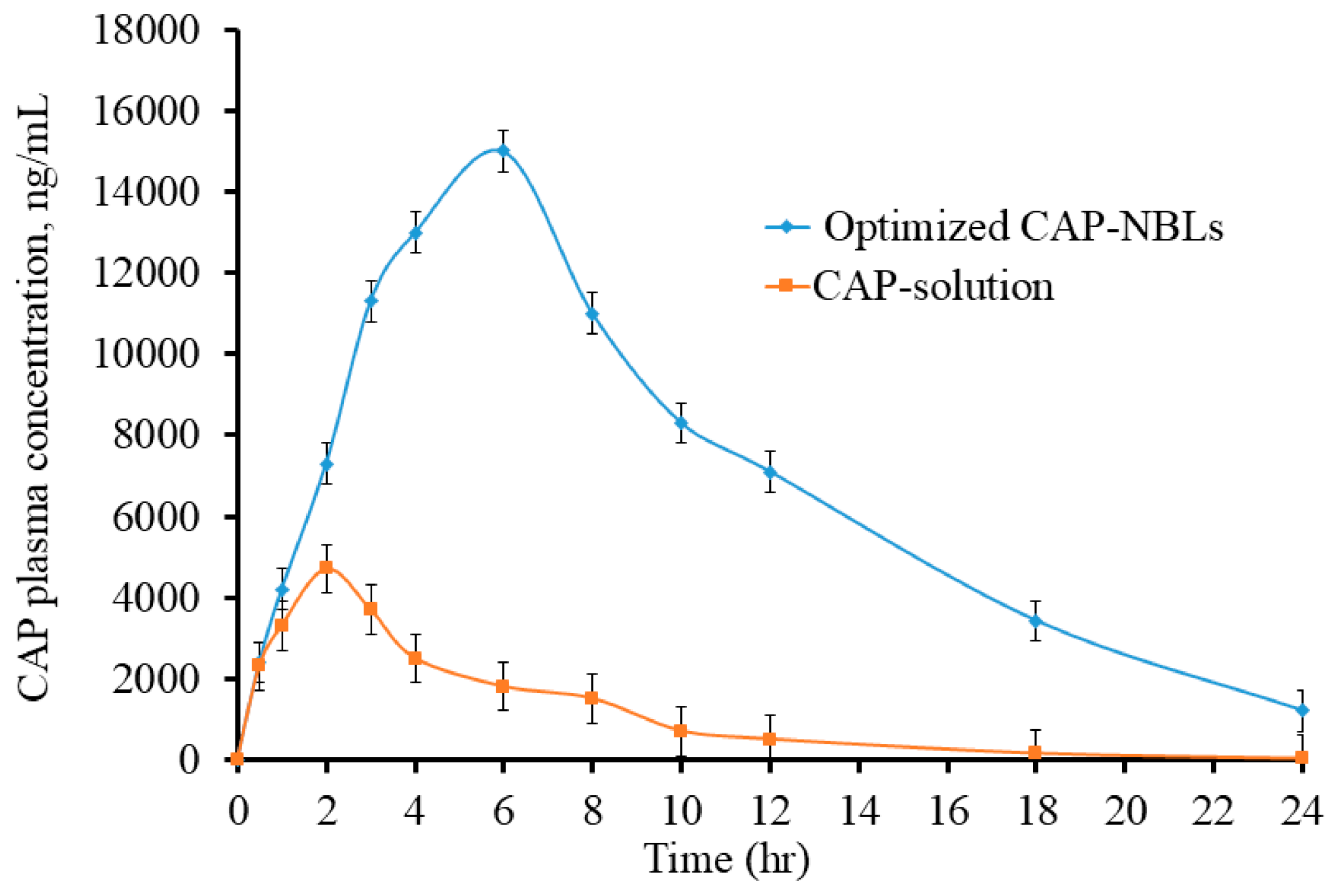
Plasma concentration against time after administration of CAP-NBLs and pure CAP solution to animals.

**Figure 14:**
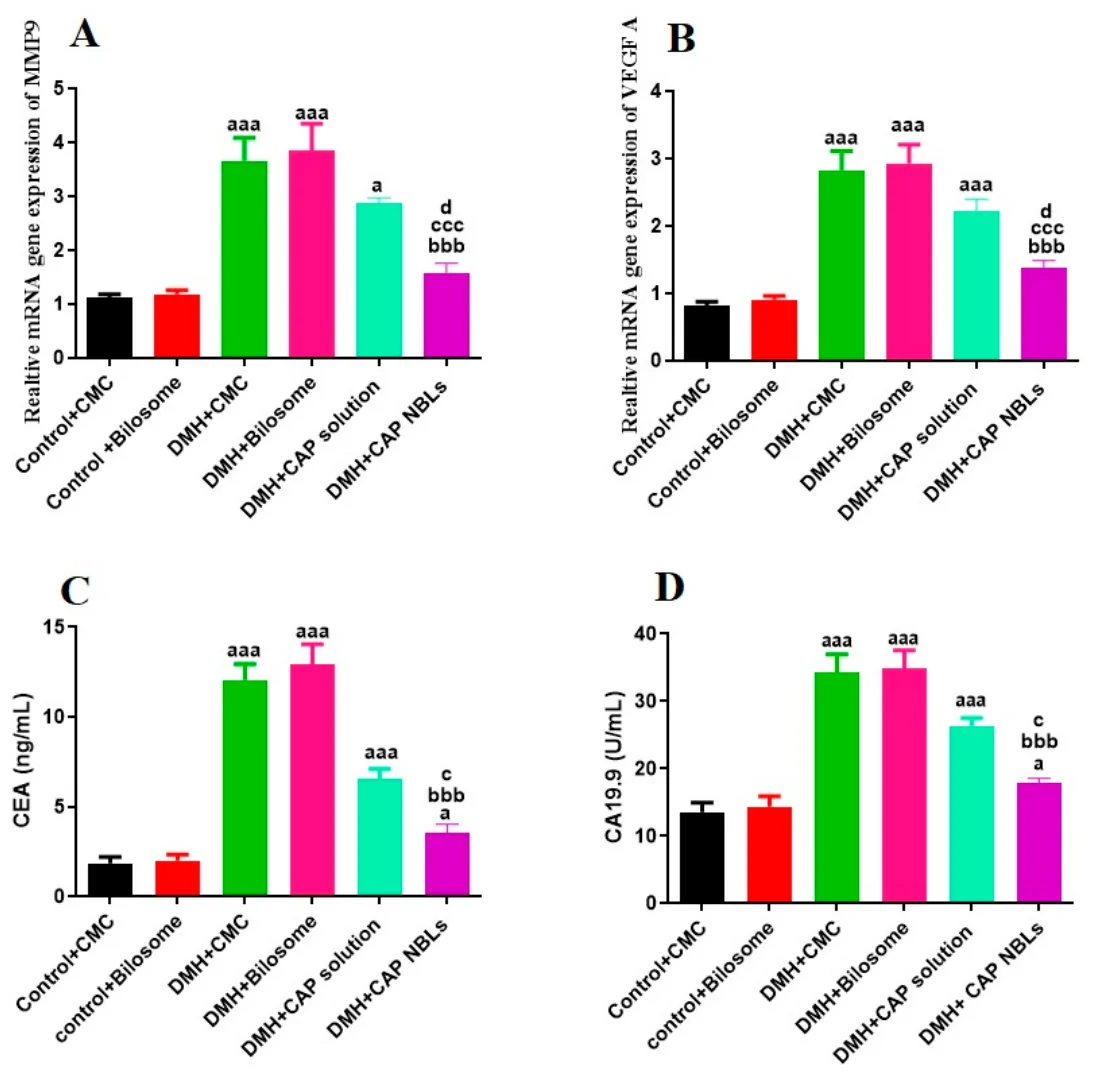
Effect of the optimized CAP-NBLs on the gene expression level of MMP-9 (**A**), VEGF-A (**B**), and CEA (**C**) and CA19.9 (**D**) serum levels in different groups. results are shown as mean ± SE (n = 12). a, b, c, and d showed a significant difference relative to the control, carcinogenic, and treated groups, respectively. a, b, c, and d (if obtained) demonstrated significant change at *p* < 0.05; aa, bb, cc, and dd (if obtained) demonstrated significant change at *p* < 0.01, aaa, bbb, ccc, and ddd (if obtained) demonstrated significant change at *p* < 0.001.

**Figure 15:**
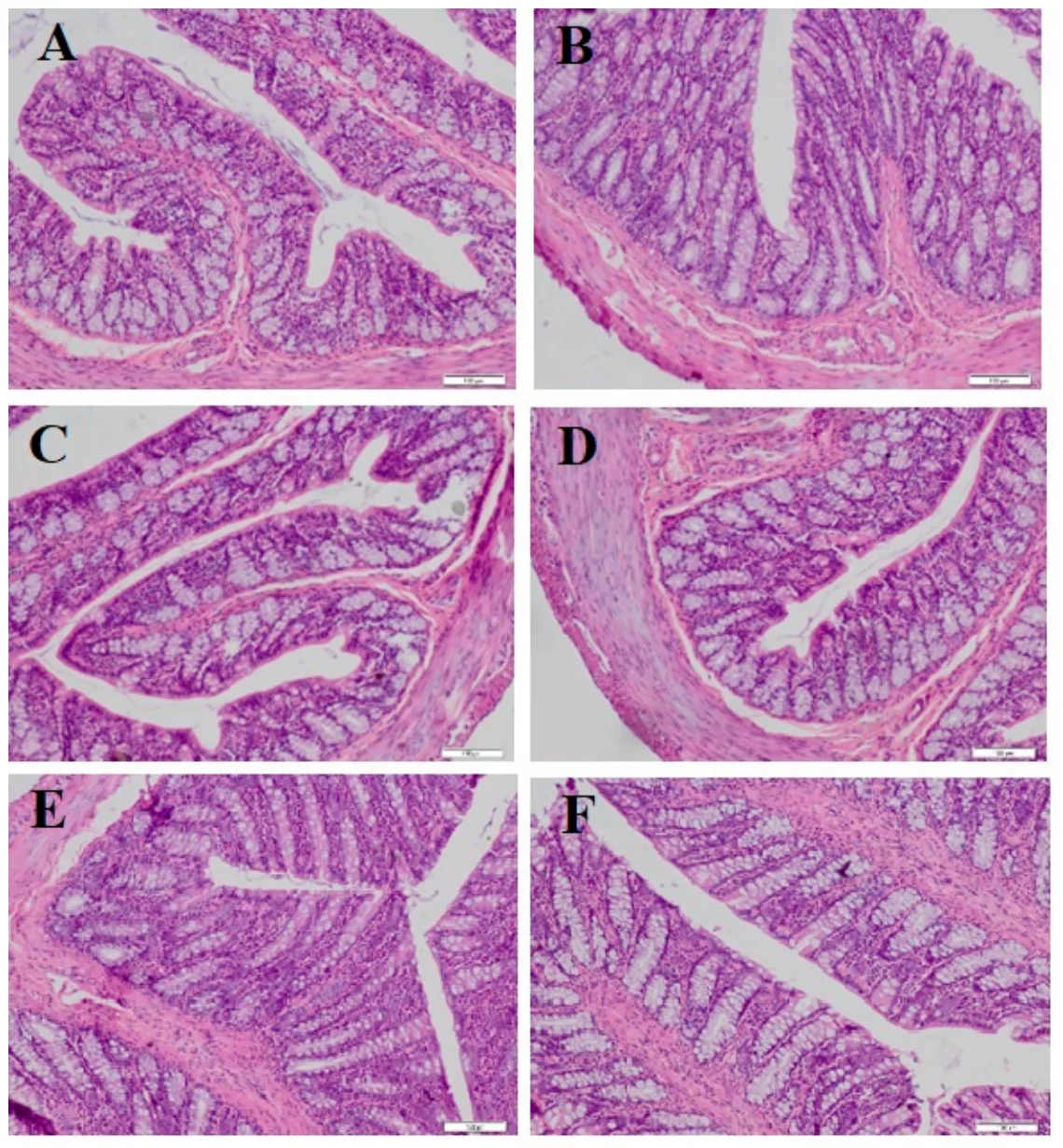
(**A**,**B**) The colon section from normal rats (groups 1 and 2), normal histoarchitecture; (**C**,**D**) the colon section from animals in groups 3 and 4, showing marked epithelial lining and crypt dysplasia; (**E**) the colon section from animals in group 5, showing decreased epithelial lining and crypt dysplasia; (**F**) the colon section from animals in group 6, displaying some restoration of colonic features with a lesser extent of epithelial lining and crypt dysplasia; scale bar, 100 µm.

**Table 1:** Characterization parameters of the various CAP-NBLs.

Run Code	CAP Content, (%)	VS (nm)	PDI	ZP (mV)
CAPB1	85.56 ± 2.58	390.23 ± 4.55	0.243	−33.4 ± 1.51
CAPB2	89.48 ± 1.11	388.3 ± 3.27	0.139	−28.9 ± 3.78
CAPB3	90.67 ± 3.39	299.1 ± 2.26	0.381	−31.6 ± 1.92
CAPB4	91.06 ± 2.71	335.7 ± 1.78	0.422	−35.5 ± 2.33
CAPB5	92.59 ± 3.22	277.32 ± 4.59	0.260	−33.8 ± 2.21
CAPB6	93.98 ± 4.12	322.1 ± 2.31	0.353	−34.5 ± 1.08
CAPB7	95.22 ± 2.09	220.32 ± 1.39	0.271	−37.1 ± 2.18
CAPB8	96.00 ± 3.26	412.45 ± 3.59	0.221	−36.7 ± 1.35
APB9	98.2 ± 3.02	370.44 ± 1.27	0.338	−35.9 ± 4.27

**Table 2:** Kinetic analysis of CAP-NBLs release data.

F. Code	Zero-Order, R^2^	First-Order, R^2^	Higuchi Diffusion, R^2^	Korsmeyer–Peppas		Fitted-Model
				R^2^	n	
CAPB1	0.877861	0.231352	0.996992671	0.985153	0.39091711	Higuchi diffusion
CAPB2	0.877534	0.239254	0.994897661	0.984449	0.402202321	Higuchi diffusion
CAPB3	0.922573	0.263472	0.991695885	0.990564	0.51890948	Higuchi diffusion
CAPB4	0.905532	0.260639	0.989901048	0.987431	0.415700914	Higuchi diffusion
CAPB5	0.92976	0.265369	0.995379464	0.993423	0.557861977	Higuchi diffusion
CAPB6	0.910972	0.263204	0.993672226	0.990114	0.46313082	Higuchi diffusion
CAPB7	0.93838	0.267769	0.992308714	0.990719	0.642869214	Higuchi diffusion
CAPB8	0.882732	0.214552	0.997750464	0.98594	0.370924654	Higuchi diffusion
CAPB9	0.862747	0.254317	0.988279739	0.977716	0.415257335	Higuchi diffusion

**Table 3:** Optimized factor level combination achieving maximum desirability based on response surface methodology.

Factor	Low	High	Optimum
Span 60, mg	75	125	125
SDC, mg	10	40	10
Response			Optimum
VS, nm			244.18
EE, %			89.41
Cumulative % drug released at 24 h			44.15

SDC: sodium deoxycholate; EE: entrapment efficiency.

**Table 4:** Permeability parameters of CAP from the optimized CAP-NBL formulation versus CAP solution (n = 3, mean ± SD).

Measured Parameter	CAP Solution	CAP-NBLs
Total permeated amount of CAP (µg)	2930 ± 57.50	7200 ± 36.10
Cumulative % amount of CAP permeated (%)	29.3 ± 2.25	72.0 ± 1.42
Permeation flux value (J_max_, µg/cm^2^/h)	2.44 ± 1.89	6 ± 1.78
APC, cm/min	2.44 × 10^−4^	6 × 10^−4^
ER	-	2.44

**Table 5:** PKPs of CAP in the optimized CAP-NBLs formulation against CAP solution (n = 3, mean ± SD).

PKPs	CAP Solution	CAP-NBLs
C_max_ (ng/mL)	4700 ± 105.12	15,000 ± 272.48
t_max_ (h)	2 ± 0.1	6 ± 0.11
AUC_0–∞_ (ng × h/mL)	27,703.86 ± 542.32	165,230.99 ± 616.35
AUMC_0–∞_ (ng × h^2^/mL)	163,042.73 ± 444.44	1,488,673.92 ± 333.56
MRT (h)	5.885 ± 0.54	9.009 ± 0.67
t_1/2_ (h^−1^)	2.92 ± 0.32	5.00 ± 0.43
Relative bioavailability (RB, %)	-	596.4

**Table 6:** 3^2^ Factorial layout CAP-NBLs.

Variable Code	Name of Variable		Level of Variable			Response Name			Aim
			−1	0	+1				
A	Span 60 (mg)		75	100	125	Y_1_: EE, %			Maximization
						Y_2_: Vesicle size, nm.			Minimization
B	SDC (mg)		10	15	20	Y_3_: Cumulative % drug amount released at 24 h, %			Minimization
Run code	CAP, mg	PC, mg	Chol, (mg)	Span 60, (mg)		SDC, (mg)	Y_1_ (%) ± SD	Y_2_ (nm) ± SD	Y_3_ (%) ± SD
CBLS1	10	100	25	75		25	63.44 ± 1.222	390.23 ± 4.55	59.5 ± 4.23
CBLS2	10	100	25	75		10	65.77 ± 2.31	388.3 ± 3.27	57.33 ± 3.67
CBLS3	10	100	25	125		40	83.21 ± 2.56	299.1 ± 2.26	48.2 ± 3.87
CBLS4	10	100	25	100		25	77.42 ± 2.34	335.7 ± 1.78	51.2 ± 2.35
CBLS5	10	100	25	125		25	85.33 ± 1.34	277.32 ± 4.59	47.33 ± 2.69
CBLS6	10	100	25	100		10	80.67 ± 2.03	322.1 ± 2.31	49.32 ± 1.34
CBLS7	10	100	25	125		10	87.66 ± 3.45	220.32 ± 1.39	45.56 ± 3.18
CBLS8	10	100	25	75		40	59.44 ± 2.34	412.45 ± 3.59	63.4 ± 3.26
CBLS9	10	100	25	100		40	73.45 ± 2.35	370.44 ± 1.27	53.31 ± 3.46

CAP: capecitabine, PC: phosphatidyl choline, Chol: cholesterol, SDC: sodium deoxycholate.

## Data Availability

The datasets generated during and/or analyzed during the current investigation are available from the corresponding authors on request.

## Supplementary Materials

The following supporting information can be downloaded at: https://www.mdpi.com/article/10.3390/ph19101531/s1, Figure S1: CAP in-vitro release profile from the optimized CAP-NBLs compared to the pure drugs in simulated gastric fluid (SGF); Table S1: Factorial design and the responses of CAP-NBLs; Table S2: ANOVA, model F-value, Ffit statistics, and final equation of CAP-NBLs EE; Table S3: ANOVA, model F-value, fFit statistics, and final equation of CAP-NBLs vesicle size; Table S4: ANOVA, model F-value, fFit statistics, and final equation of CAP-NBLs in vitro release after 12 h.

## Abbreviations

The following abbreviations are used in this manuscript:

5-FU	5-fluorouracil
AUC	Area under the plasma concentration–time curve
ATR-FTIR	Attenuated total reflectance-Fourier transform infrared
CEA	Cancer embryonic antigen
CAP	Capecitabine
CAP-NBLs	Capecitabine nanosized bilosomes
CA 19.9	Carbohydrate antigen 19.9
CRC	Colorectal cancer
EE%	Entrapment efficiency percentage
MMP-9	Matrix metalloproteinase-9
PC	Phosphatidylcholine
RT-PCR	Real-time polymerase chain reaction
SGF	Simulated gastric fluid
SIF	Simulated intestinal fluid
SDC	Sodium deoxycholate
VEGF	Vascular endothelial growth factor A
