# Supplementary material for: Nanosized Bilosomes as a Potential Platform for Improved Therapeutic Efficacy of Capecitabine Against Colon Cancer in Rats: Formulation, Evaluation, and Optimization
Source: Pharmaceuticals (Basel). 2026 Sep 27;19(10):1531. doi: 10.3390/ph19101531 (PMC13708600; doi:10.3390/ph19101531)
Supplement: Supplementary file 1 [file pharmaceuticals-19-01531-s001.zip › pharmaceuticals-4492046-supplementary.pdf]

Supplementary materials

Title: Nanosized Bilosomes as a Potential Platform for Improved Therapeutic Efficacy of Capecitabine against colon cancer in rats: Formulation, Evaluation, and Optimization

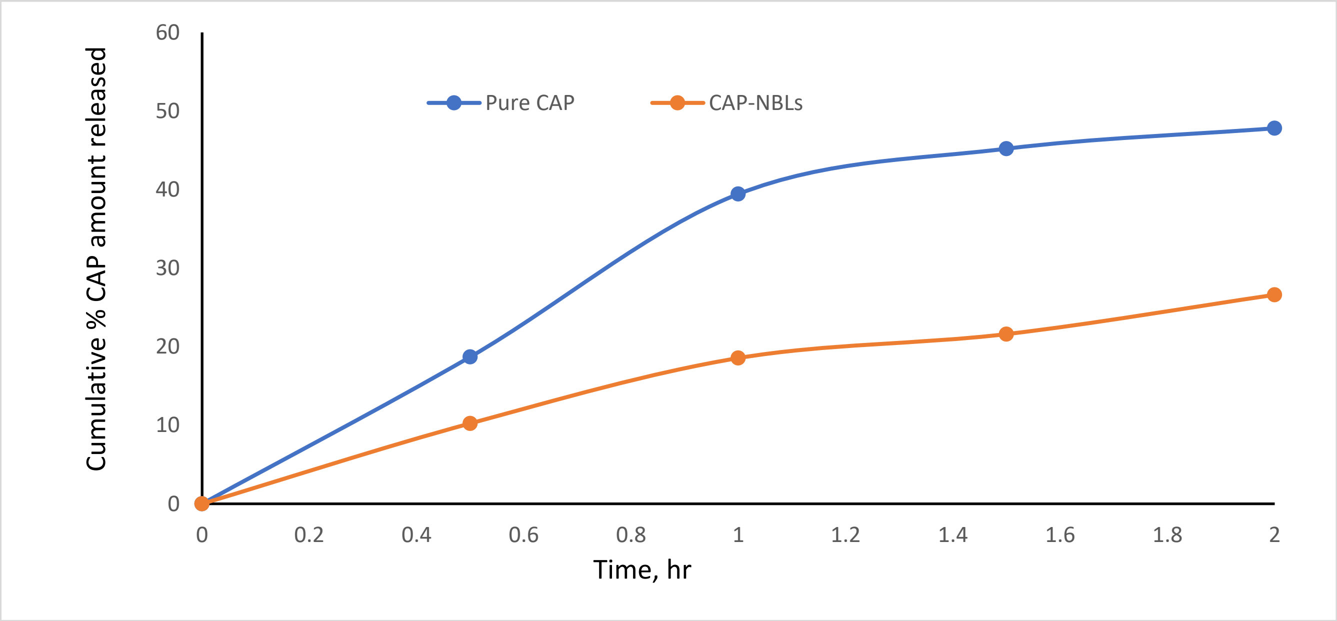

Fig.S1. CAP in-vitro release profile from the optimized CAP-NBLs compared to the pure drugs in simulated gastric fluid (SGF)

Optimization Data of CAP-NBLs

Table S1. Factorial design and the responses of CAP-NBLs

Run	Factor 1 A:Span 60 mg	Factor 2 B:SDC mg	Response 1 EE %	Response 2 VS nm	Response 3 Rel 24h
1	75	25	63.44	390.23	59.5
2	75	10	65.77	388.3	57.33
3	125	40	83.21	299.1	48.2
4	100	25	77.42	335.7	51.2
5	125	25	85.33	277.32	47.33
6	100	10	80.67	322.1	49.32
7	125	10	87.66	220.32	45.56
8	75	40	59.44	412.45	63.4
9	100	40	73.45	370.44	53.31

Table S2. ANOVA, model F-value, Fit statistics, and final equation of CAP-NBLs EE

Configure | Fit Summary | Model | ANOVA | Diagnostics | Model Graphs

Analysis of Variance

ANOVA for Linear model

Response 1: EE

Source	Sum of Squares	df	Mean Square	F-value	p-value	
**Model**	814.50	2	407.25	116.24	< 0.0001	significant
A-Span 60	760.50	1	760.50	217.06	< 0.0001	
B-SDC	54.00	1	54.00	15.41	0.0077	
**Residual**	21.02	6	3.50			
**Cor Total**	835.52	8				

Factor coding is **Coded.**
Sum of squares is **Type III - Partial**

The **Model F-value** of 116.24 implies the model is significant. There is only a 0.01% chance that an F-value this large could occur due to noise.

P-values less than 0.0500 indicate model terms are significant. In this case A, B are significant model terms. Values greater than 0.1000 indicate the model terms are not significant. If there are many insignificant model terms (not counting those required to support hierarchy), model reduction may improve your model.

Fit Statistics | Model Comparison Statistics

Fit Statistics

**Std. Dev.**	1.87	**R^2**	0.9748
**Mean**	75.15	**Adjusted R^2**	0.9665
**C.V. %**	2.49	**Predicted R^2**	0.9455
		**Adeq Precision**	26.3879

The **Predicted R^2** of 0.9455 is in reasonable agreement with the **Adjusted R^2** of 0.9665; i.e. the difference is less than 0.2.

[illegible]

Coefficients | Coded Equation | Actual Equation

Final Equation in Terms of Actual Factors

EE	=
+35.12111	
+0.450333	* Span 60
-0.200000	* SDC

The equation in terms of actual factors can be used to make predictions about the response for given levels of each factor. Here, the levels should be specified in the original units for each [illegible]

Table S3. ANOVA, model F-value, Fit statistics, and final equation of CAP-NBLs vesicle size

Analysis of Variance

ANOVA for Linear model

Response 2: VS

Source	Sum of Squares	df	Mean Square	F-value	p-value	
**Model**	29717.97	2	14858.98	65.46	< 0.0001	significant
A-Span 60	25904.20	1	25904.20	114.13	< 0.0001	
B-SDC	3813.77	1	3813.77	16.80	0.0064	
**Residual**	1361.87	6	226.98			
**Cor Total**	31079.83	8				

Factor coding is **Coded**.
Sum of squares is **Type III - Partial**

The **Model F-value** of 65.46 implies the model is significant. There is only a 0.01% chance that an F-value this large could occur due to noise.

P-values less than 0.0500 indicate model terms are significant. In this case A, B are significant model terms. Values greater than 0.1000 indicate the model terms are not significant. If there are many insignificant model terms (not counting those required to support hierarchy), model reduction may improve your model.

Fit Statistics | Model Comparison Statistics

Fit Statistics

**Std. Dev.**	15.07	**R^2**	0.9562
**Mean**	335.11	**Adjusted R^2**	0.9416
**C.V. %**	4.50	**Predicted R^2**	0.8762
		**Adeq Precision**	20.9050

The **Predicted R^2** of 0.8762 is in reasonable agreement with the **Adjusted R^2** of 0.9416; i.e. the difference is less than 0.2.

[illegible]

Coefficients | Coded Equation | Actual Equation

Final Equation in Terms of Actual Factors

VS	=
+555.91389	
-2.62827	* Span 60
+1.68078	* SDC

The equation in terms of actual factors can be used to make predictions about the response for given levels of each factor. Here, the levels should be specified in the original units for each [illegible]

Table S4. ANOVA, model F-value, Fit statistics, and final equation of CAP-NBLs invitro release after 12 hrs

Analysis of Variance

ANOVA for Linear model

Response 3: Rel 24h

Source	Sum of Squares	df	Mean Square	F-value	p-value	
**Model**	282.20	2	141.10	60.50	0.0001	significant
A-Span 60	255.32	1	255.32	109.47	< 0.0001	
B-SDC	26.88	1	26.88	11.53	0.0146	
**Residual**	13.99	6	2.33			
**Cor Total**	296.20	8				

Factor coding is **Coded.**
Sum of squares is **Type III - Partial**

The **Model F-value** of 60.50 implies the model is significant. There is only a 0.01% chance that an F-value this large could occur due to noise.

P-values less than 0.0500 indicate model terms are significant. In this case A, B are significant model terms. Values greater than 0.1000 indicate the model terms are not significant. If there are many insignificant model terms (not counting those required to support hierarchy), model reduction may improve your model.

Fit Statistics | Model Comparison Statistics

Fit Statistics

**Std. Dev.**	1.53	**R^2**	0.9528
**Mean**	52.79	**Adjusted R^2**	0.9370
**C.V. %**	2.89	**Predicted R^2**	0.8887
		**Adeq Precision**	19.5974

The **Predicted R^2** of 0.8887 is in reasonable agreement with the **Adjusted R^2** of 0.9370; i.e. the difference is less than 0.2.

[illegible]

Coefficients | Coded Equation | Actual Equation

Final Equation in Terms of Actual Factors

Rel 24h	=
+75.36000	
-0.260933	* Span 60
+0.141111	* SDC

The equation in terms of actual factors can be used to make predictions about the response for given levels of each factor. Here, the levels should be specified in the original units for each [illegible]
